# CDS2 expression regulates *de novo* phosphatidic acid synthesis

**DOI:** 10.1042/BCJ20240456

**Published:** 2024-10-11

**Authors:** Daniel M. Collins, Vishnu Janardan, David Barneda, Karen E. Anderson, Izabella Niewczas, Diane Taylor, Danye Qiu, Henning J. Jessen, Andrea F. Lopez-Clavijo, Simon Walker, Padinjat Raghu, Jonathan Clark, Len R. Stephens, Phillip T. Hawkins

**Affiliations:** 1 Signalling Programme, Babraham Institute, Cambridge CB22 3AT, U.K.; 2 National Centre for Biological Sciences-TIFR GKVK Campus, Bangalore, India; 3 Institute of Organic Chemistry, University of Freiburg, Albertstr. 21, 79104 Freiburg, Germany

**Keywords:** CDP-DG, CDS2, lipidomics, metabolism, phosphatidic acids, phosphatidylinositol

## Abstract

CDS enzymes (CDS1 and 2 in mammals) convert phosphatidic acid (PA) to CDP-DG, an essential intermediate in the *de novo* synthesis of PI. Genetic deletion of CDS2 in primary mouse macrophages resulted in only modest changes in the steady-state levels of major phospholipid species, including PI, but substantial increases in several species of PA, CDP-DG, DG and TG. Stable isotope labelling experiments employing both ^13^C_6_- and ^13^C_6_D_7_-glucose revealed loss of CDS2 resulted in a minimal reduction in the rate of *de novo* PI synthesis but a substantial increase in the rate of *de novo* PA synthesis from G3P, derived from DHAP via glycolysis. This increased synthesis of PA provides a potential explanation for normal basal PI synthesis in the face of reduced CDS capacity (via increased provision of substrate to CDS1) and increased synthesis of DG and TG (via increased provision of substrate to LIPINs). However, under conditions of sustained GPCR-stimulation of PLC, CDS2-deficient macrophages were unable to maintain enhanced rates of PI synthesis via the ‘PI cycle’, leading to a substantial loss of PI. CDS2-deficient macrophages also exhibited significant defects in calcium homeostasis which were unrelated to the activation of PLC and thus probably an indirect effect of increased basal PA. These experiments reveal that an important homeostatic response in mammalian cells to a reduction in CDS capacity is increased *de novo* synthesis of PA, likely related to maintaining normal levels of PI, and provides a new interpretation of previous work describing pleiotropic effects of CDS2 deletion on lipid metabolism/signalling.

## Introduction

Cytidine diphosphate diacylglycerol synthases (CDSs) catalyse the formation of cytidine diphosphate diacylglycerol (CDP-DG) and pyrophosphate (PPi) from phosphatidic acid (PA) and CTP. The CDP-DG synthesised by CDS enzymes in the ER is the substrate for phosphatidylinositol synthase and thus a key intermediate in the biosynthesis of phosphatidylinositol (PI) and more highly phosphorylated phosphoinositides [phosphatidylinositol monophosphates (PIP; PI3P; PI4P; PI5P); phosphatidylinositol bisphosphates (PIP2: PI(4,5)P2; PI(3,4)P2; PI(3,5)P2) and phosphatidylinositol trisphosphate (PIP3: PI(3,4,5)P3) [1]. Phosphoinositides are established to play critical roles in signal transduction, membrane identity and intracellular trafficking in all eukaryotic cells [2–4]. CDP-DG synthesised by TAM41 in the mitochondria is thought to supply most of the substrate for phosphatidylglycerol phosphate (PGP), phosphatidylglycerol (PG) and cardiolipin (CL) synthesis [5], but this partition between the CDP-DG synthesised by CDSs and TAM41 may not be absolute.

**Figure 1.**
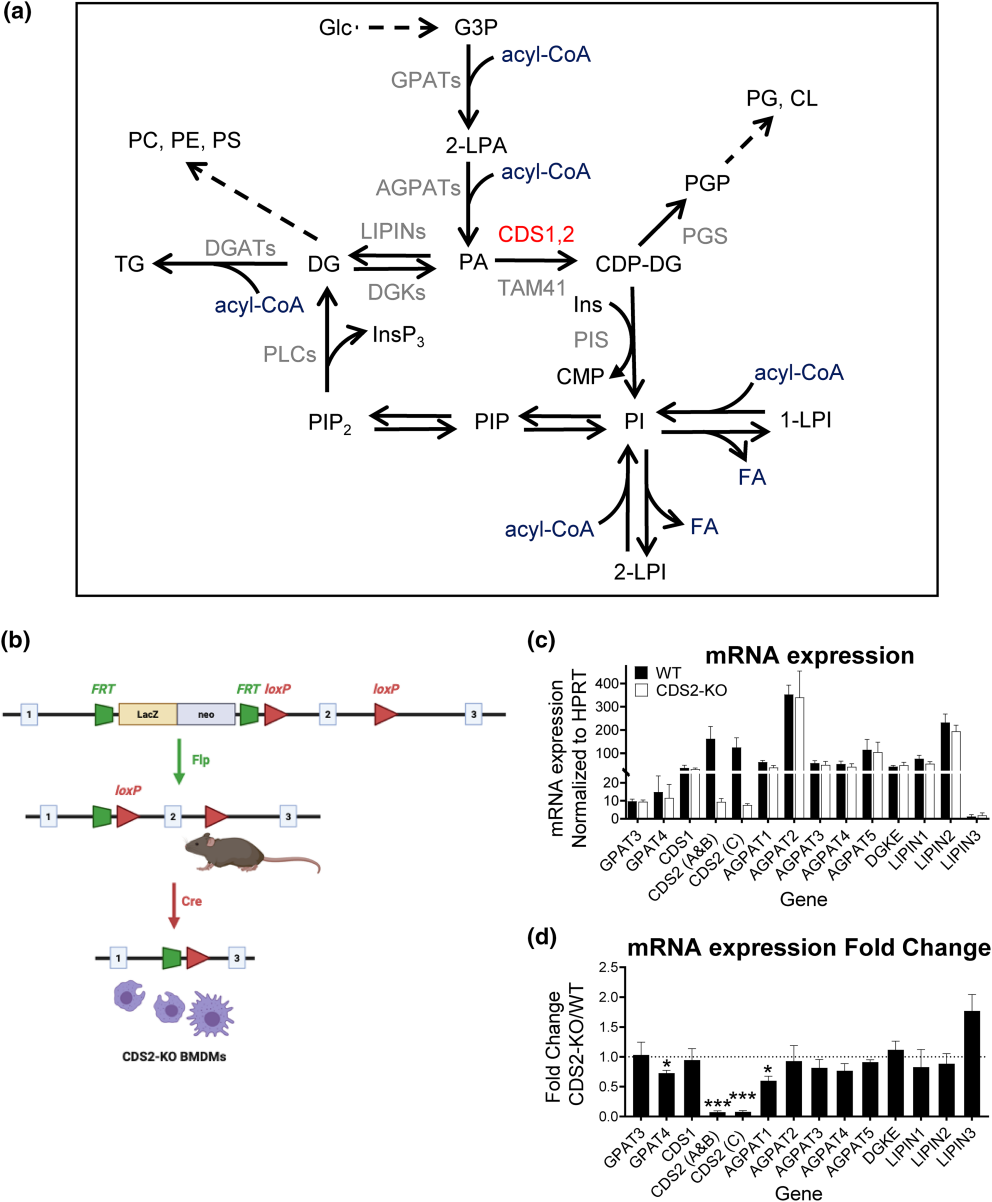
Creation of CDS2-KO mouse macrophages. (**a**) The position of CDS2 in the metabolic pathways of lipid biosynthesis. (**b**) A schematic overview of the generation of CDS2-KO mouse macrophages. CDS2-targetted embryonic stem cells were purchased from the UCDAVIS KOMP Repository (KOMP ES cell line *Cds2^tm1a(KOMP)Wtsi^*). Mice generated from these stem cells were first crossed with mice expressing FLPe, resulting in the excision of the LacZ-neo selection cassette. The offspring of this cross were then further crossed with LysM-Cre-expressing mice, resulting in the myeloid-selective deletion of exon-2 of the CDS2 gene and subsequent generation of a frameshift mutation during bone marrow differentiation into mature macrophages. (**c**) qRT-PCR analysis of the mRNA levels for relevant genes in bone marrow-derived macrophages. CDS2 (A,B and C) refer to different potential transcripts of the CDS2 gene, as described in Supplementary Figure S1. Values for mRNA expression were normalised to the HPRT gene and are represented as means ± S.E.M (*n *= 3). (**d**) mRNA levels represented as fold changes between WT and CDS2-KO macrophages. Statistical significance was assessed using one sample *t*-test of three independent biological experiments (*n* = 3) and represented as mean ± S.E.M (*P <* *0.05, **P <* *0.01, ***P <* *0.001).

The substrate for CDSs, PA, is also a substrate for PA phosphatase (LIPIN)-mediated dephosphorylation to diacylglycerol (DG), providing the biosynthetic substrate for the formation of other phospholipid classes [phosphatidylcholine (PC), phosphatidylethanolamine (PE), phosphatidylserine (PS) and triacylglycerol (TG)]. PA is synthesised *de novo* by the successive acylation of glycerol 3-phosphate (G3P), catalysed by glycerol phosphate acyltransferase (GPAT) and acylglycerol phosphate acyltransferase (AGPAT) activities, and thus the conversion of PA to either CDP-DG or DG represents a major branch point in *de novo* lipid biosynthesis (Figure 1a). This branch point would be assumed to integrate multiple feedback regulatory loops to ensure the cell has the appropriate relative complement of lipid pools. However, whilst much is known about the regulation of LIPIN isoforms, particularly in the control of TG synthesis [6–8], much less is known about how CDS enzymes are co-ordinated with *de novo* lipid biosynthesis.

Lower eukaryotes possess a single CDS activity that is essential for life [1]. In flies, eye-specific deletion of CDS confirms an essential role for CDS activity in supporting the resynthesis of PI subsequent to receptor-stimulated hydrolysis of PI(4,5)P2 by phospholipase C (PLC) (known as the ‘PI-cycle’) [9]. Higher eukaryotes, including mammals, possess two related CDS activities (CDS1 and 2) [1]. Several studies have investigated the consequences of reducing the levels of each isoform individually and these have revealed partially overlapping but distinct roles. CDS2 is more widely expressed and, in some contexts at least, appears to play the major role in the receptor-stimulated PI-cycle; a property that may be dependent on both its more peripheral ER distribution and its greater preference for the stearoyl/arachidonoyl species of PA (this is the predominant acyl chain species of phosphoinositides in most mammalian cells) [1,10–12].

Some of the phenotypes associated with loss of CDS activity have been linked to reductions in PI and a consequent impact on PLC and phosphoinositide 3-kinase (PI3K) signalling pathways, though often without clarity over the observed reductions in PI(4,5)P2 (relative to the kinetic parameters of PLC/PI3K) necessitated by such explanations [13,14]. Loss of CDS activity is also generally reported to cause an expected increase in the levels of its substrate, PA e.g. depletion of CDS in flies results in an elevation of PA levels without a major change in the steady state levels of PI [15]. PA has many roles in regulating membrane biology, as well as being a key biosynthetic intermediate, and thus increases in PA are likely to have pleiotropic effects, many of which will be less obviously linked to a direct effect on CDS activity [16–18]. A common observation is that loss of CDS activity causes an increase in TG and an accumulation of super-sized lipid droplets [13,19–21]. In some cases, this is accompanied by paradoxically small decreases in PI levels, rendering simple explanations based on ‘re-directed flux’ from CDSs to LIPINs more difficult.

To get a clearer picture of the effects of reducing CDS activity on the rates of PI and PA synthesis, we investigated the effects of deleting CDS2 on lipid metabolism in primary mouse macrophages using mass spectrometry and a stable isotope labelling approach. Our results uncover a new connection between CDSs and *de novo* PA synthesis.

## Results

### Creation of mouse macrophages lacking CDS2

Our strategy for LysMcre-mediated deletion of CDS2 in the myeloid compartment of the mouse is shown in Figure 1b. Primary bone marrow-derived macrophages (BMDM) from these mice (CDS2-KO) and their wild-type (WT) controls were then analysed for the expression of several lipid biosynthetic enzymes of interest. We could not detect CDS2 mRNA in CDS2-KO macrophages by RT-PCR (Figure 1c) and substantial deletion of CDS2 protein was confirmed by Western blot (Supplementary Figure S1). The level of CDS1 mRNA was unaffected by deletion of CDS2, but we did observe modest reductions in the levels of mRNA encoding AGPAT1 and GPAT4 ( ∼25–40%) and a possible increase in mRNA encoding LIPIN3 (Figure 1c,d). Furthermore, supporting previous observations following loss of CDS activity [13,19–21], we observed increased accumulation of lipid droplets in macrophages derived from CDS2-KO mice (Supplementary Figure S2).

### The effect of CDS2 deletion on the steady-state levels of major lipid classes

**Figure 2.**
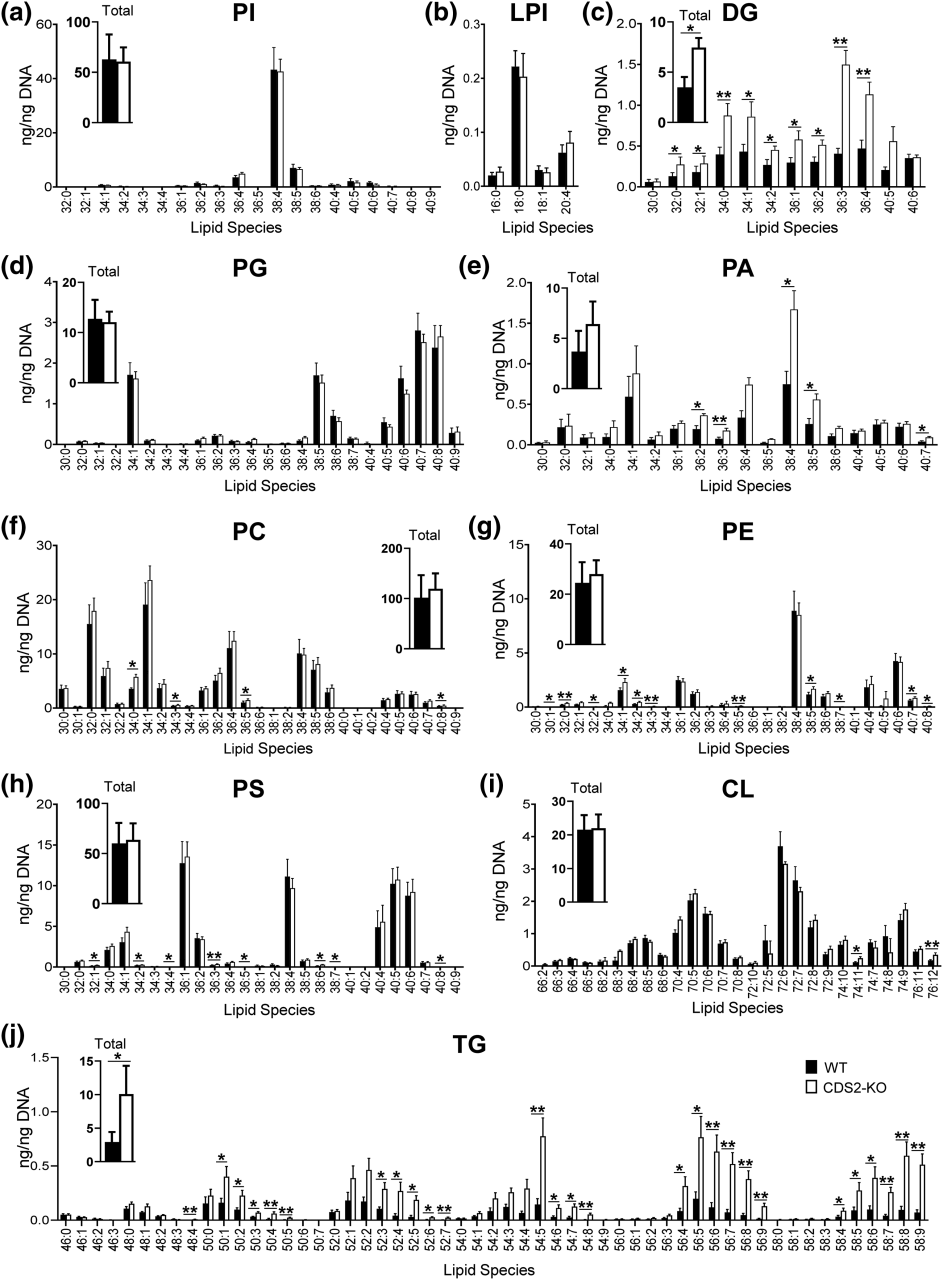
A broad overview of the steady-state levels of major lipid species in WT and CDS2-KO macrophages. PI, DG, PG, PA, PC, PE, PS, CL, and TG molecular species in WT (black filled bars) or CDS2-KO (open bars) macrophages were analysed by untargeted mass spectrometry using an Orbitrap coupled to liquid chromatography (LC–MS). The total levels for the lipid subclasses measured are included as insets in the relevant panels. LPI was analysed using a Triple Quad coupled to liquid chromatography (LC–MS/MS). Data is presented in nanograms (ng) of lipid (calculated as the ratio of the area of each molecular species to the area of a known quantity of internal standard) normalised to the DNA concentration in the initial cellular extract and represent means ± S.E.M (*n* = 4). Statistical significance was assessed using paired student's *t*-test for total lipid levels and ratio-paired student's *t*-test for individual molecular species of four independent biological experiments (*P < 0.05, **P < 0.01).

We analysed the effects of CDS2 deletion on the basal lipidome of mouse macrophages grown in serum/GM-CSF. We first employed high resolving power-high mass accuracy untargeted mass spectrometry using an Orbitrap Elite coupled to liquid chromatography (Orbitrap LC–MS) to measure the molecular species of the major lipid subclasses present in these cells. Loss of CDS2 had remarkably little effect on most of the lipid molecular species measured (Figure 2), including a surprisingly negligible impact on PI 38:4 (Figure 2a; 38:4 represents the combined total number of carbons : total number of double bonds in both acyl chains). However, we did observe significant increases in some molecular species of PA (e.g. PA 38:4; Figure 2e) and multiple molecular species of DG (Figure 2c) and TG (particularly in those species containing poly-unsaturated fatty acids; Figure 2j) in CDS2-KO cells. These increases in DG and TG were reflected in 2–4-fold increases in the total levels of all molecular species measured for these two lipid classes (Figure 2c,j insets).

**Figure 3.**
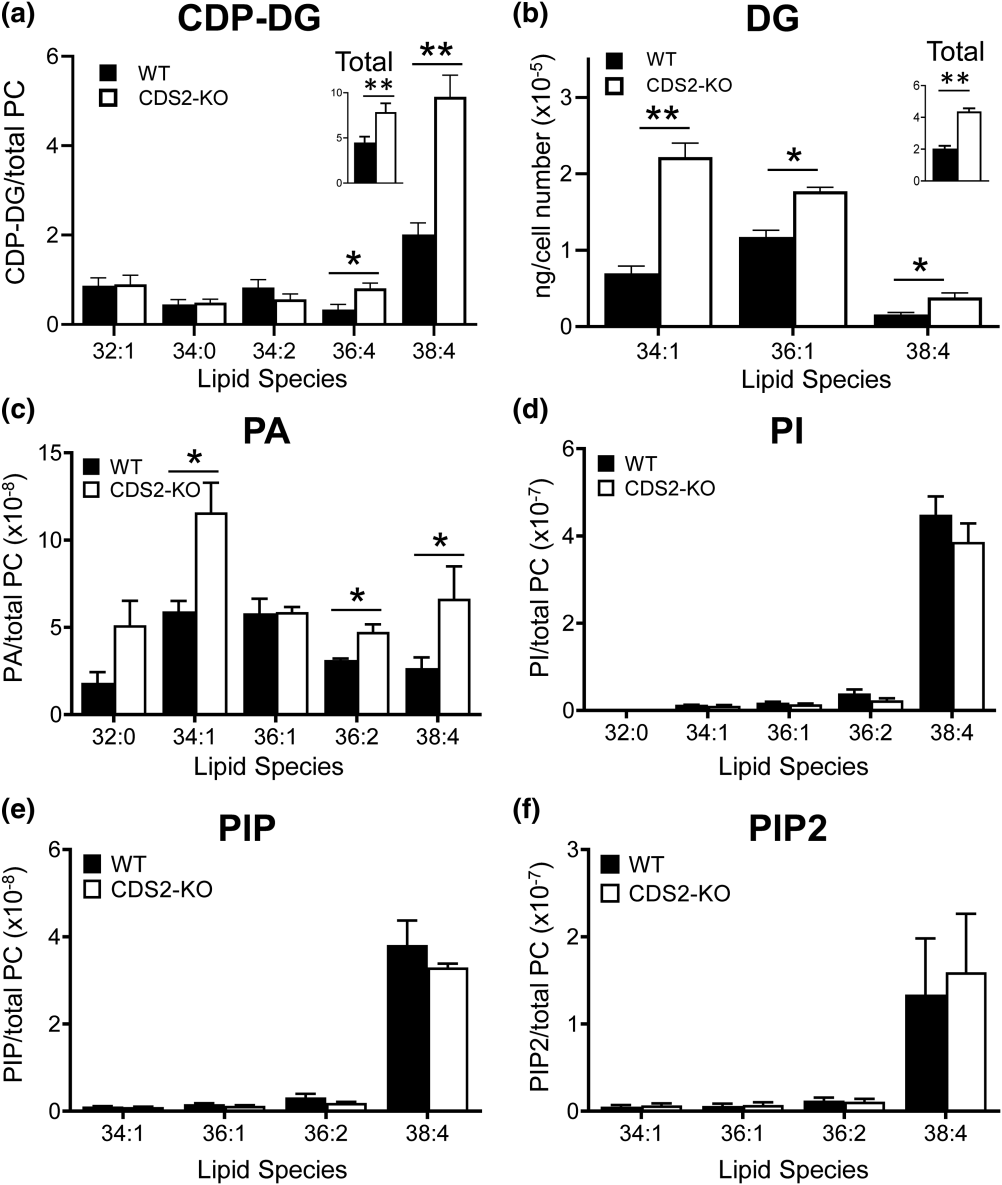
A targeted mass spectrometry analysis of selected lipid species in WT and CDS2-KO macrophages. The indicated lipid species in WT or CDS2-KO macrophages were analysed by targeted mass spectrometry using a Triple Quad coupled to liquid chromatography (LC–MS/MS). Data are either response ratios (peak areas corrected for the recovery of the internal standard) normalised to peak areas for total PC (for PA, PI, PIP, PIP2; trial experiments indicated this was the most robust normalisation method to compare different WT and CDS2-KO macrophage cultures derived from independent mice, see Supplementary Figure S3; *n *= 3), or peak areas normalised to peak areas for total PC (for CDP-DG; no internal standard for CDP-DG was available; *n *= 4), or ng corrected to cell number (for DG; ng calculated from a standard curve using known amounts of DG standard; PC was not measured in these lipid extracts; *n *= 3) and are means ± S.E.M. Statistical significance was assessed using a ratio-paired student's *t*-test (*P < 0.05, **P < 0.01).

We confirmed and extended this Orbitrap analysis for selected lipid species using previously established targeted mass spectrometry methods (Triple Quad LC–MS/MS). This enabled superior quantitation of some molecular species and the additional measurement of PIP, PIP2 and CDP-DG (Figure 3). This confirmed the minimal impact of CDS2 deletion on phosphoinositide levels (Figure 3d–f) and also the substantial increases in DG (Figure 3b) and PA (Figure 3c). Remarkably, the loss of CDS2 did not result in the expected decrease in levels of CDP-DG, but a significant increase (particularly in the 38:4 species; Figure 3a). PC, PE and PS levels were also confirmed to not change significantly with the loss of CDS2 (Supplementary Figure S3).

### The effect of CDS2 deletion on the *de novo* synthesis of PA and PI

**Figure 4.**
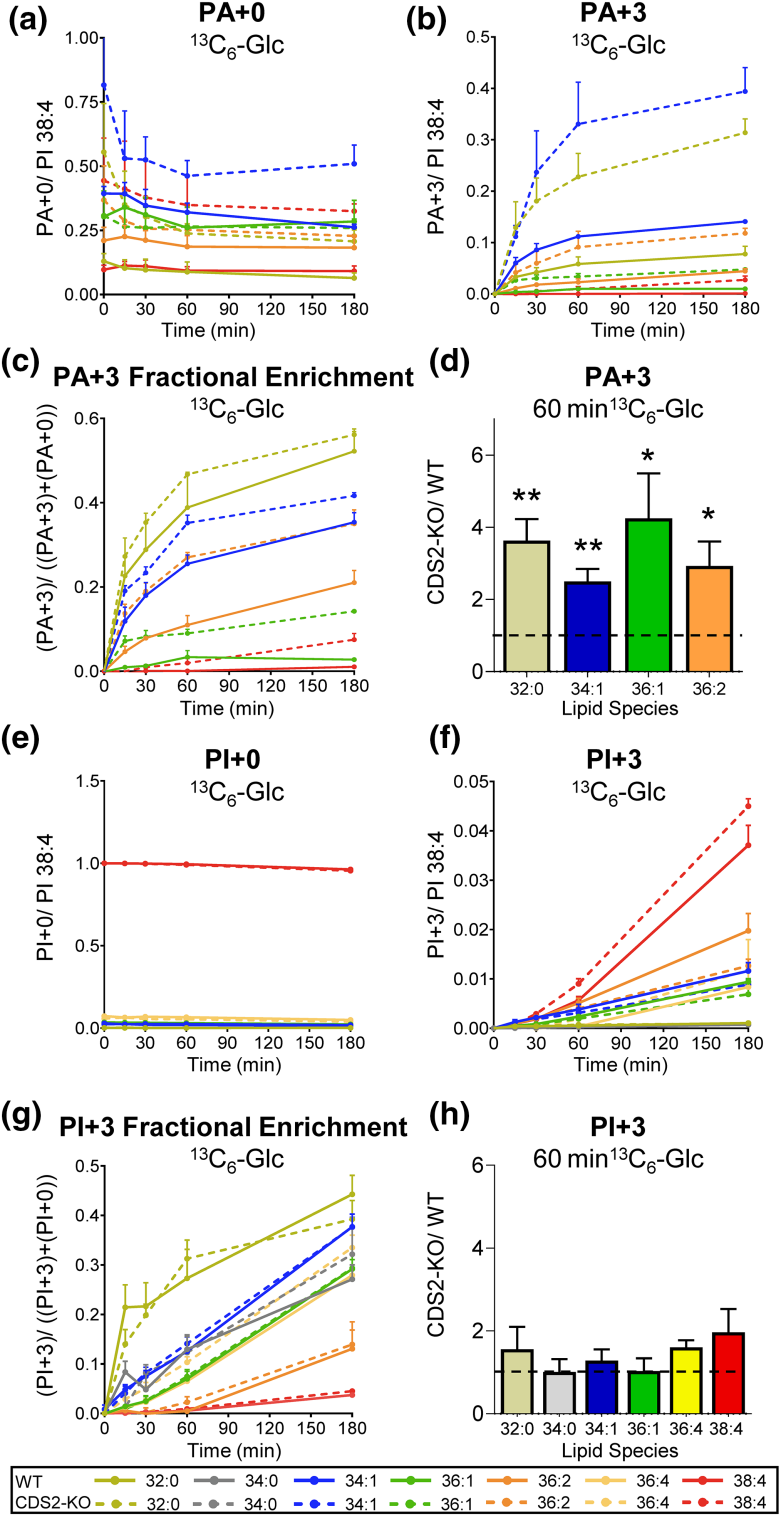
The rates of *de novo* synthesis of PA and PI in WT and CDS2-KO macrophages assessed by labelling with ^13^C_6_-Glc. ^13^C_6_-Glucose was added at *t *=* *0 and the incorporation of ^13^C_3_ (+3 Da) into the glycerol backbone of the major PA and PI species in WT and CDS2-KO macrophages was analysed by LC–MS/MS at the times indicated. Data for the indicated isotopologues (+0* *=* *unlabelled; +3* *=* *labelled) are response ratios (peak areas corrected to the recovery of the PI internal standard) normalised to the levels of PI 38:4 at *t *=* *0 and represent means* *±* *S.E.M (*n *=* *3). ‘Fractional enrichment’ is defined as the ratio of the indicated +3-isotopologue divided by the sum of labelled and unlabelled isotopologues. Statistical significance was assessed using a one sample *t*-test (*n *=* *3–9) (*P <* *0.05, **P <* *0.01).

We then interrogated potential changes in the rates of the underlying biosynthetic pathways which could result in the changes to steady-state lipid levels observed in CDS2-KO macrophages. PA is synthesised *de novo* by the acylation of glycerol 3-phosphate (G3P) synthesised via glycolysis (Figure 1a). We tracked the rate of *de novo* synthesis of PA by measuring the initial rate of incorporation of ^13^C nuclei from ^13^C_6_-glucose into the glycerol backbone of multiple PA species (Figure 4a–d). The most prominent accumulations of ^13^C_3_ into PA (PA + 3) were observed in shorter chain, more saturated molecular species (PA 32:0; PA 34:1), which we and others have described as characteristic of the immediate products of *de novo* PA synthesis [10]. In contrast, initial accumulation of PA + 3 38:4 was much lower (Figure 4b).

The accumulations of several PA + 3 species were much larger in CDS2-KO macrophages compared with WT ( ∼3–4-fold; Figure 4d). Furthermore, the fractional enrichment of the +3-species (a measure of the turnover of the entire pool at steady state) was similar or larger in CDS2-KO macrophages compared with the WT, for each of the PA-species measured (Figure 4c). This indicates that the larger pool sizes of several different PA species in CDS2-KO cells (particularly PA 34;1; see above) are served by substantially increased flux both in and out of these pools (because over the time course of these experiments, these pools are at steady-state).

PI is synthesised *de novo* via PA and CDP-DG (Figure 1a). Our LC–MS/MS methods did not have the sensitivity to measure the accumulation of the ^13^C_3_-labelled glycerol backbone in CDP-DG, but we were able to measure the accumulation of several species of ^13^C_3_-labelled PI (PI + 3) (Figure 4e–h). The pattern of accumulation of PI + 3 species was very similar to that we have reported previously, there is a faster fractional turnover of the smaller pools of shorter chain, more saturated species (e.g. PI 32:0, PI 34:1) and a slower fractional enrichment of the much larger pool of PI 38:4 (Figure 4g) [10]. We have shown previously that this pattern is generated by the rapid conversion of PA species synthesised *de novo* into PI, which then undergo rapid acyl chain remodelling to form the larger pool of PI 38:4 [10]. We observed similar rates of PI + 3 accumulation between CDS2-KO and WT macrophages (e.g. PI + 3 38:4; Figure 4h) and near identical rates of fractional enrichment for the smaller, more rapidly turned over pools (e.g. PI 34:1) (Figure 4g).

### The origin of the effect of CDS2 deletion on *de novo* PA synthesis

**Figure 5.**
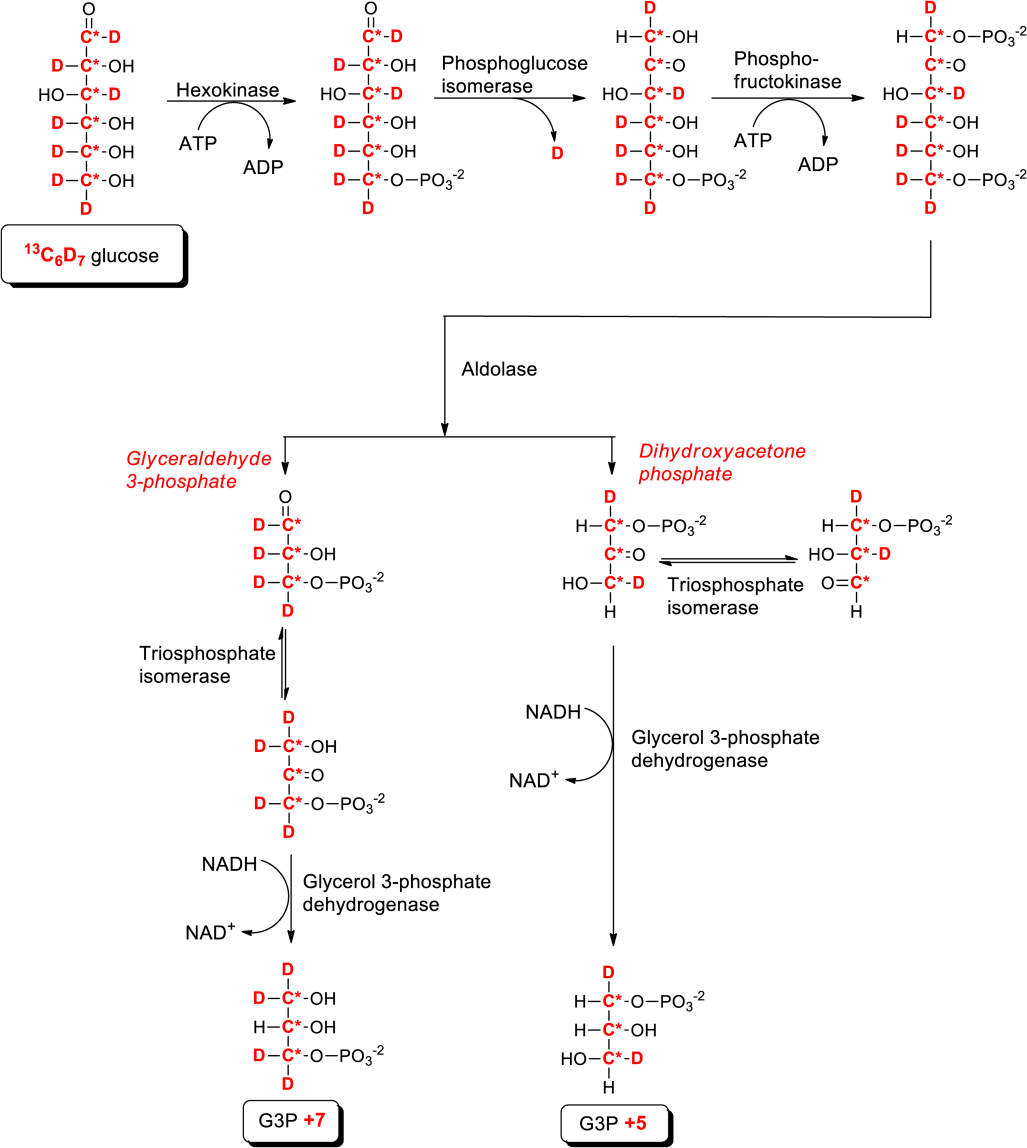
Proposed biosynthetic pathways for the incorporation of heavy isotopes from ^13^C_6_D_7_-Glucose into triose phosphates. The potential metabolic pathways for incorporation of ^13^C and ^2^H (D) nuclei (labelled in red) from ^13^C_6_D_7_-glucose into glycerol 3-phosphate via glycolysis.

We sought to improve the signal-to-noise for our measurements of heavy isotope incorporation into newly synthesised PA and PI species by using ^13^C_6_D_7_-glucose, which enables the incorporation of further deuterium nuclei into the glycerol backbone used for PA synthesis (Figure 5). In addition, the use of ^13^C_6_D_7_-glucose provided further information on the metabolic route taken for PA synthesis; the asymmetric cleavage of fructose (1,6)-bisphosphate by aldolase into glyceraldehyde 3-phosphate (Gly3P) and dihydroxyacetone 3-phosphate (DHAP) leads to glycerol 3-phosphate (G3P) labelled with either ^13^C_3_D_2_ (+5 Da) or ^3^C_3_D_4_ (+7 Da), depending on which of the two triosephosphates generated by glycolysis acted as its source (Figure 5).

**Figure 6.**
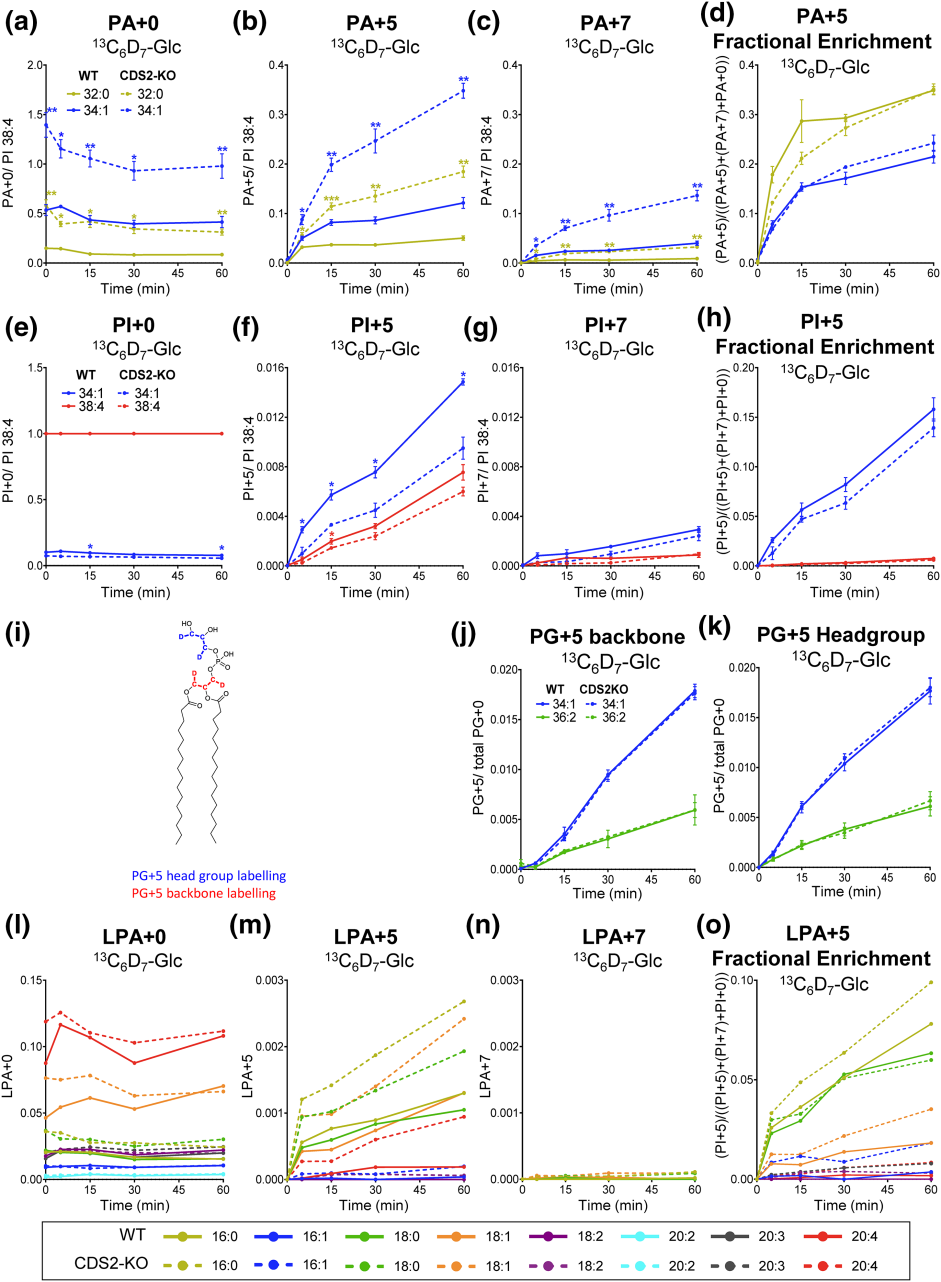
The rates of *de novo* synthesis of PA, PI, LPA and PG in WT and CDS2-KO macrophages assessed by labelling with ^13^C_6_D_7_-Glc. ^13^C_6_D_7_-glucose was added at *t *=* *0 and the incorporation of either +5 or +7 Da into the indicated isotopologues in WT and CDS2-KO macrophages were analysed by LC–MS/MS at the times indicated. Data are response ratios (peak areas corrected for the recovery of the relevant PA, PI, or LPA internal standards) normalised to PI 38:4 at *t *=* *0 (**a–c, e–g, l,m**), or peak areas (for PG, no PG internal standard was available) normalised to total PG at *t *=* *0 (**j,k**) and represent means* *±* *S.E.M (*n *=* *3–4). ‘Fractional enrichment’ is defined as the ratio of the indicated +3-isotopologue divided by the sum of labelled and unlabelled isotopologues. Statistical significance was assessed using multiple paired *t*-test (*P <* *0.05, **P <* *0.01). Diagram (i) illustrates the locations of ^13^C and ^2^H (D) incorporation (from G3P; see Figure 5) into the PG backbone and headgroup.

We present data describing the isotopic incorporation of ^13^C_3_D_2_ (+5) or ^3^C_3_D_4_ (+7) into the two main species of PA synthesised *de novo* in macrophages (PA 32:0, PA 34:1; Figure 6a–d, Supplementary Figure S4a,b). We observed much greater accumulation of PA + 5 species than PA + 7 species ( ∼3 fold; Figure 6b vs c), indicating DHAP is the main source G3P used for PA synthesis. Furthermore, this data confirmed the substantially greater rate of *de novo* PA synthesis in CDS2-KO macrophages compared with the WT that we observed with ^13^C_6_-glucose labelling ( ∼3-fold; Figures 4b and 6b).

We also measured the accumulation of ^13^C_3_D_2_-PI (PI + 5) and ^13^C_3_D_4_-PI (PI + 7) molecular species in these experiments (Figure 6e–h, Supplementary Figure S4c,d). The +5/+7 ratio for the PI 34:1 species was similar to that seen for PA 34:1 ( ∼4-fold vs 5-fold, respectively) consistent with their precursor product relationship. The rate of accumulation of +5/+7-labelled PI 34:1 was slightly greater in WT vs CDS2-KO macrophages, but with more similar rates of fractional accumulation (Figure 6f–h), possibly reflecting slight differences in the PI 34:1 complements of the two genotypes in this set of experiments compared with those used for the experiments presented in Figure 4. Overall, however, these experiments clearly support a substantial increase in *de novo* PA synthesis in CDS2-KO macrophages relative to a much smaller effect on *de novo* PI synthesis.

We also measured the rate of ^13^C_6_D_7_-glucose labelling of PG in these experiments. PG is synthesized by dephosphorylation of PGP which, in turn, is synthesized from CDP-DG and G3P in a reaction catalysed by PGP synthase (PGS; Figure 1a). The rate of labelling of both the glycerol backbone and glycerol headgroup were almost identical between WT and CDS2-KO cells (Figure 6i–k, Supplementary Figure S4e,f). This indicates loss of CDS2 does not affect the rate of production of G3P via glycolysis/Gly3P-dehydrogenase and also confirms the lack of any substantial effect on the rate of production of PG.

This data suggested loss of CDS2 stimulates *de novo* synthesis at a step between the production of G3P and the formation of PA. G3P is acylated successively at the *sn-1* and *sn-2* positions by GPAT and AGPAT activities (Figure 1a). We therefore also measured the incorporation of isotope label from ^13^C_6_D_7_-glucose into several species of LPA in WT and CDS2-KO macrophages (Figure 6l–o). We observed a substantial increase in the rate of accumulation of ^13^C_3_D_2_-LPA (LPA + 5) species in CDS2-KO cells with the potential to represent intermediates in the production of PA 32:0 and PA 34:1 (LPA + 5 16:0; LPA + 5 18:0 and LPA + 5 18:1; Figure 6m). Combined with the lack of effect of CDS2-deletion on the labelling of G3P, this is strong evidence that loss of CDS2 stimulates the step catalysed by GPAT activity.

### The effect of CDS2 deletion on total synthesis of PA and PI under basal conditions

**Figure 7.**
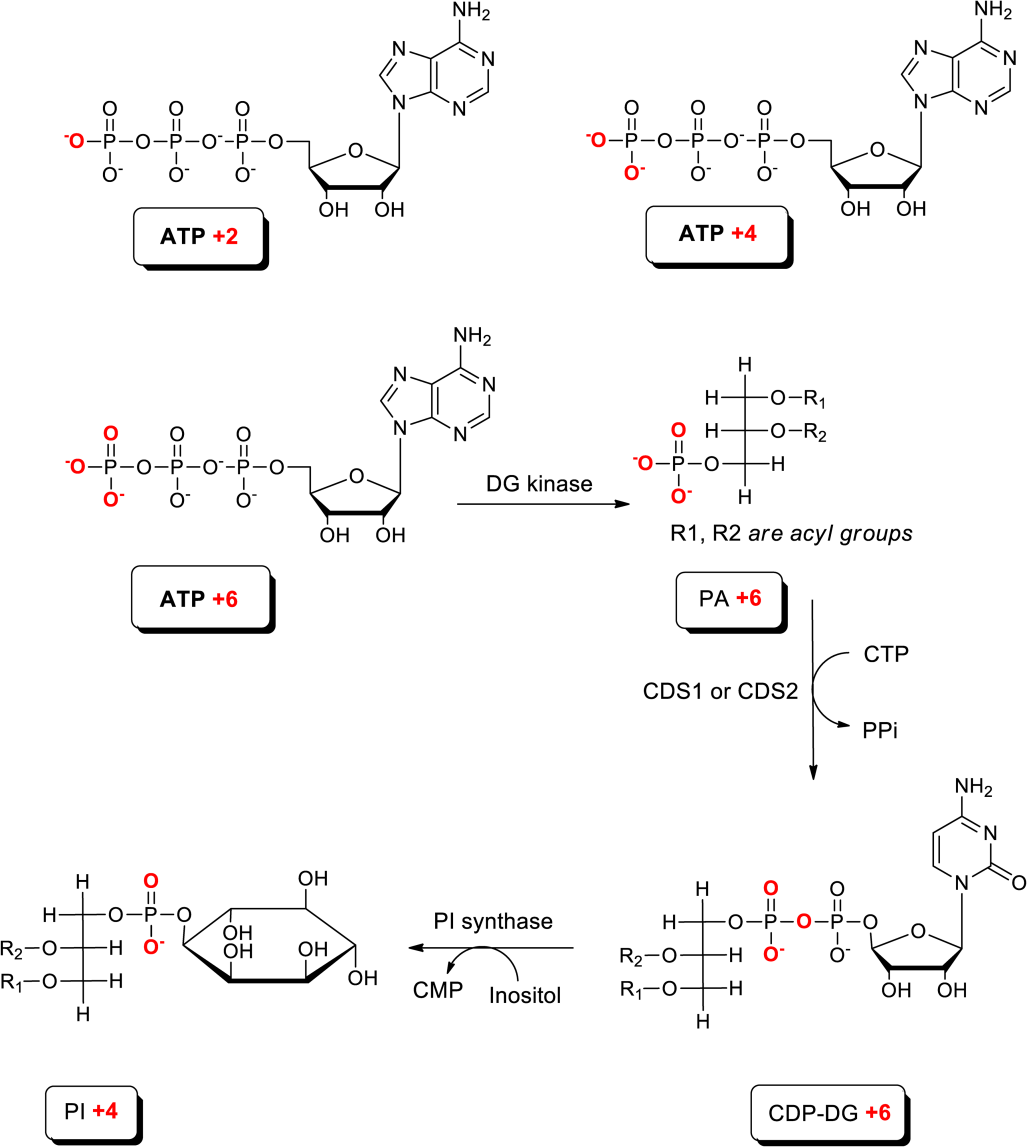
Proposed pathways for incorporation of heavy isotopes from H_2_^18^O into PA and PI. The potential metabolic pathways for incorporation of ^18^O nuclei (labelled in red) from H_2_^18^O into PA and PI. Rapid phosphotransferase reactions in the cell lead to the random incorporation of ^18^O nuclei into the non-bridging oxygens (ie those oxygens not in the phosphate anhydride linkage) in the gamma-phosphate of ATP, to generate ATP+2, +4, or +6 isotopologues. The utilization of these isotopologues of ATP then generates the requisite isotopologues of PA (+2/+4/+6), CDP-DG (+2/+4/+6) and PI (+2/+4); only the utilization of ATP+6 is shown for clarity. In practice, the different potential isotopologues of PA and PI behaved very similarly in our experiments but only data for PA+4 and PI+4 are presented because they represented the most robust measurements with the least signal/noise.

In addition to *de novo* synthesis, PA can be synthesized from pre-existing DG through a reaction catalysed by DG-kinase (DGK; Figure 1a). We have previously shown that an estimate of the rate at which new phosphate groups are incorporated into PA (via either glycolysis or DGK) can be obtained by measuring the rate at with ^18^O-nuclei are transferred to PA, via the nucleotide pool, from ^18^O-labelled water added to the culture medium (see Figure 7) [10].

**Figure 8.**
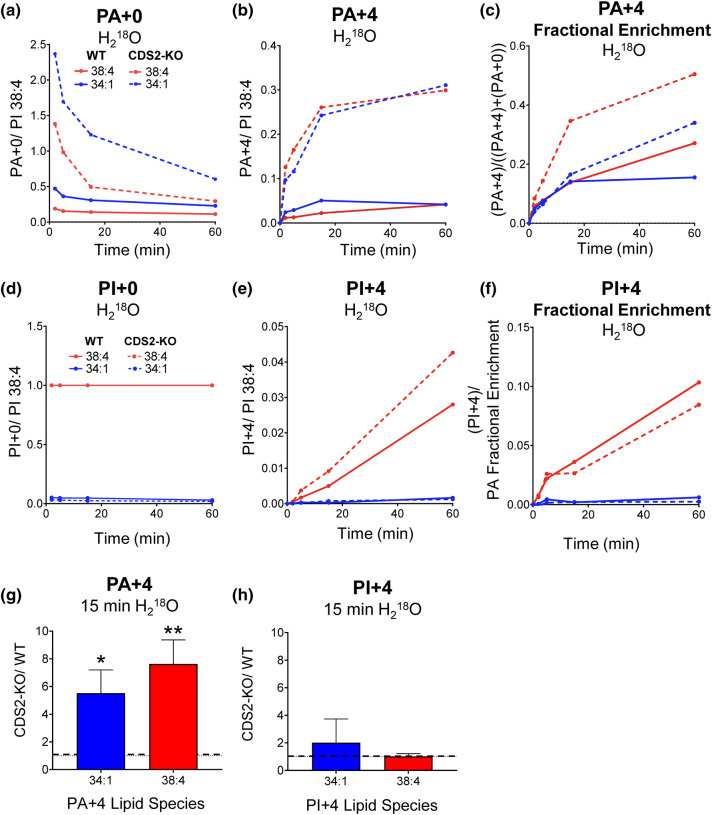
The rates of total synthesis of PA and in WT and CDS2-KO macrophages assessed by labelling with H_2_^18^O. H_2_^18^O was added at *t *=* *0 and the incorporation of ^18^O_2_ (+4 Da) into the 34:1-and 38:4-species of PA and PI in WT or CDS2-KO macrophages were analysed by LC–MS/MS at the times indicated. Data are response ratios (peak areas corrected for the recovery of the relevant PA or PI internal standards) normalised to PI 38:4 at *t *=* *0 and show one representative experiment of three that produced similar results (**a,b, d,e**). ‘Fractional Enrichment’ for PA is defined as the ratio of the +4-isotopologue divided by the sum of labelled and unlabelled isotopologues (**c**) or ‘Fractional Enrichment’ for PI is defined as the ratio of the +4-isotopologue divided by the ‘PA Fractional Enrichment’ (to correct for the change in precursor PA labelling between WT and CDS2-KO (**f**). Fold change of PA+4 (**g**) and PI+4 (corrected for ‘PA Fractional Enrichment’; (**h**) at 15 min labelling represent means ± S.E.M (*n *= 7). Statistical significance was assessed using one sample *t*-test (*P <* *0.05, **P <* *0.01).

We observed very rapid and substantial accumulation of both PA + 4 38:4 and PA + 4 34:1 (Figure 8a–c) and the rates of labelling of both species were substantially increased in CDS2-KO cells compared with the WT (5–10-fold; Figure 8b). This was also seen in the enhanced fractional accumulation of label into these species (Figure 8c). A comparison with the glucose labelling data presented in Figure 4 indicates DGK activity directed against DG 38:4 must be substantially elevated in CDS2-KO cells and is consistent with previous reports of substantial 38:4-selectivity for the DGK reaction in these cells [10].

We also observed rapid accumulation of PI + 4 38:4 and a much lower accumulation of PI + 4 34:1 (Figure 8d–f). This is in line with the relative abundances of these two molecular species at steady state and consistent with previous studies indicating that most PI synthesis in these cells utilises the phosphorylation of pre-existing DG 38:4, as opposed to PA created by *de novo* synthesis (and reported by glucose-labelling, see above) [10]. The accumulation of PI + 4 species between WT and CDS2-KO cells was much more similar than for PA + 4 species, especially when the accumulation of PI + 4 was corrected for the increased fractional accumulation of PA + 4 in the CDS2-KO (Figure 8f,h).

A very similar rate of total PI synthesis between WT and CDS2-KO cells was also confirmed by measuring the incorporation of ^18^O/^2^H-inositol, which is a measure of total PI synthesis independent of the source of CDP-DG/PA (Supplementary Figure S5).

### The effect of CDS2 deletion on the UDP-stimulated PI-cycle

We investigated the effect of CDS2 deletion on phosphoinositide levels in cells stimulated with UDP. UDP is established to activate PLCβ in mouse macrophages through Gi-coupled purinergic receptors (P_2_Y_2_) [22]. The activation of PLC leads to rapid hydrolysis of PI(4,5)P2 and the production of the second messengers InsP_3_ and DG. The DG formed is rapidly converted into PA, which is then transferred to the ER via lipid exchange proteins and acts as a source for enhanced CDP-DG and PI synthesis, a process known as the ‘PI-cycle’ (Figure 1a), and which ultimately acts to maintain cellular phosphoinositide levels in the face of sustained PLC stimulation [23].

**Figure 9.**
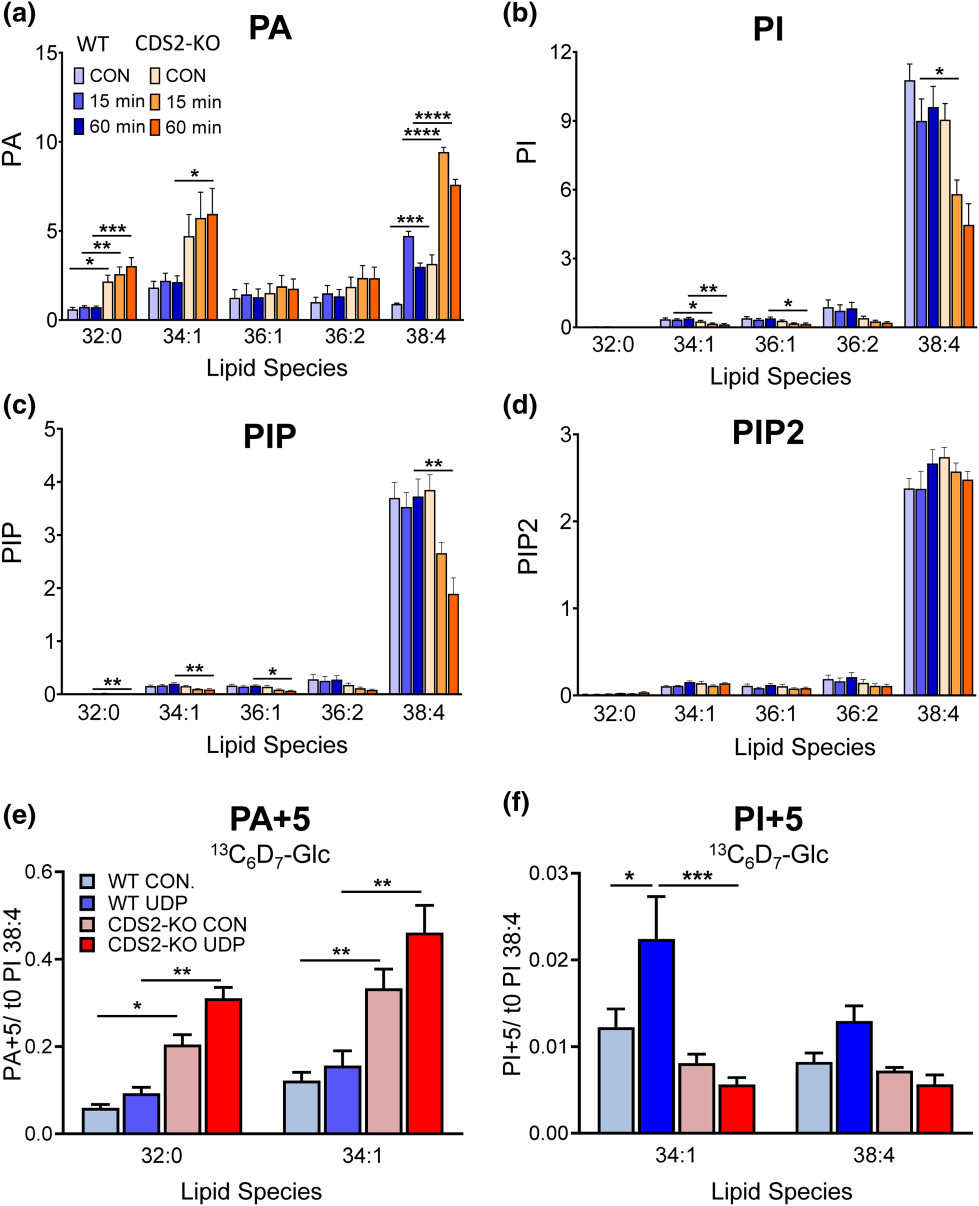
The effect of CDS2 deletion on a UDP-stimulated PI cycle. (**a–d**) WT or CDS2-KO macrophages were incubated with or without 100 µM UDP for 15 or 60 min before analysis of the major species of PA, PI, PIP and PIP2 by LC–MS/MS. Data are response ratios (peak areas corrected for the recovery of the relevant internal standards) normalised to the mean of each vehicle control for PI 38:4 (except for PA, where the *t *= 15 min UDP mean was used because it exhibited less variation between biological replicates). Statistical significance was assessed using two-way ANOVA with Šídák's test to correct for multiple comparisons of four independent biological experiments (*n *=* *4) and data represented as mean ± S.E.M. (*P < 0.05, **P < 0.01, ***P < 0.001). (**e,f**) WT or CDS2-KO macrophages were labelled with ^13^C_6_D_7_-glucose for 30 min prior to incubation with or without 100 µM UDP for 30 min and then incorporation of +5 Da into the indicated isotopologues of PA and PI in WT analysed by LC–MS/MS (data for the analogous +7 isotopologues are shown in Supplementary Figure S5). Data are response ratios (peak areas corrected for the recovery of the relevant internal standards) normalised to PI 38:4 at *t *=* *0 and represent means ± S.E.M. (*n *= 3). Statistical significance was assessed using a two-way ANOVA with Tukey's test to correct for multiple comparisons (*P <* *0.05, **P <* *0.01, ***P <* *0.001).

UDP-stimulation of WT macrophages resulted in the rapid and sustained accumulation of PA 38:4 (Figure 9a). This is the molecular species of PA that would be expected to originate (via DG and DGK) from PLC activity directed against the predominant molecular species of PI(4,5)P2 in these cells, 38:4. However, the levels of PI, PIP and PIP2 were relatively unaffected by UDP-stimulation, indicating the PI-cycle in WT macrophages is highly efficient, maintaining phosphoinositide levels during 60 min of stimulation (Figure 9b–d); this is typical of many primary cells stimulated through endogenous levels of receptors [24]. In contrast, in CDS2-KO cells, the already high basal levels of PA were enhanced still further upon stimulation (Figure 9a) and the levels of PIP and PI dropped quickly and dramatically (to ∼50% of unstimulated levels) (Figure 9b,c). Interestingly however, the total levels of PIP2 were maintained in CDS2-KO cells (Figure 9d), indicating active synthesis from the much larger pools of PI/PIP (presumably by enzymes still working above their relevant *K_m_*). This suggests that CDS2 is essential to maintain a stimulated PI-cycle in these cells.

We also investigated the rates of *de novo* PA and PI synthesis under UDP-stimulated conditions using our ^13^C_6_D_7_-glucose tracing strategy. In WT cells, UDP stimulated a small but statistically insignificant accumulation of PA + 5 32:0/34:1 (Figure 9e) and a larger, significant increase in PI + 5 34:1 (Figure 9f). This confirms an observation we made recently that GPCR-stimulation of PLC can lead to an increase in *de novo* synthesis of PI, in addition to stimulated recycling of PI through a PI-cycle [10]. In CDS2-KO cells, there was increased accumulation of PA + 5 species compared with WT cells, but we observed no stimulated increases in PI + 5 (Figure 9e,f). This suggests that there is a bottleneck in the conversion of PA to PI in UDP-stimulated CDS2-KO cells, and that the excess accumulation of unlabelled PA generated via PLC dilutes out the pool of PA + 5 generated via *de novo* synthesis, leading to reduced PI + 5 synthesis. Similar results were found for PA + 7 and PI + 7 accumulation in response to UDP (Supplementary Figure S6).

### The effect of CDS2 deletion on Ca^2+^ responses

**Figure 10.**
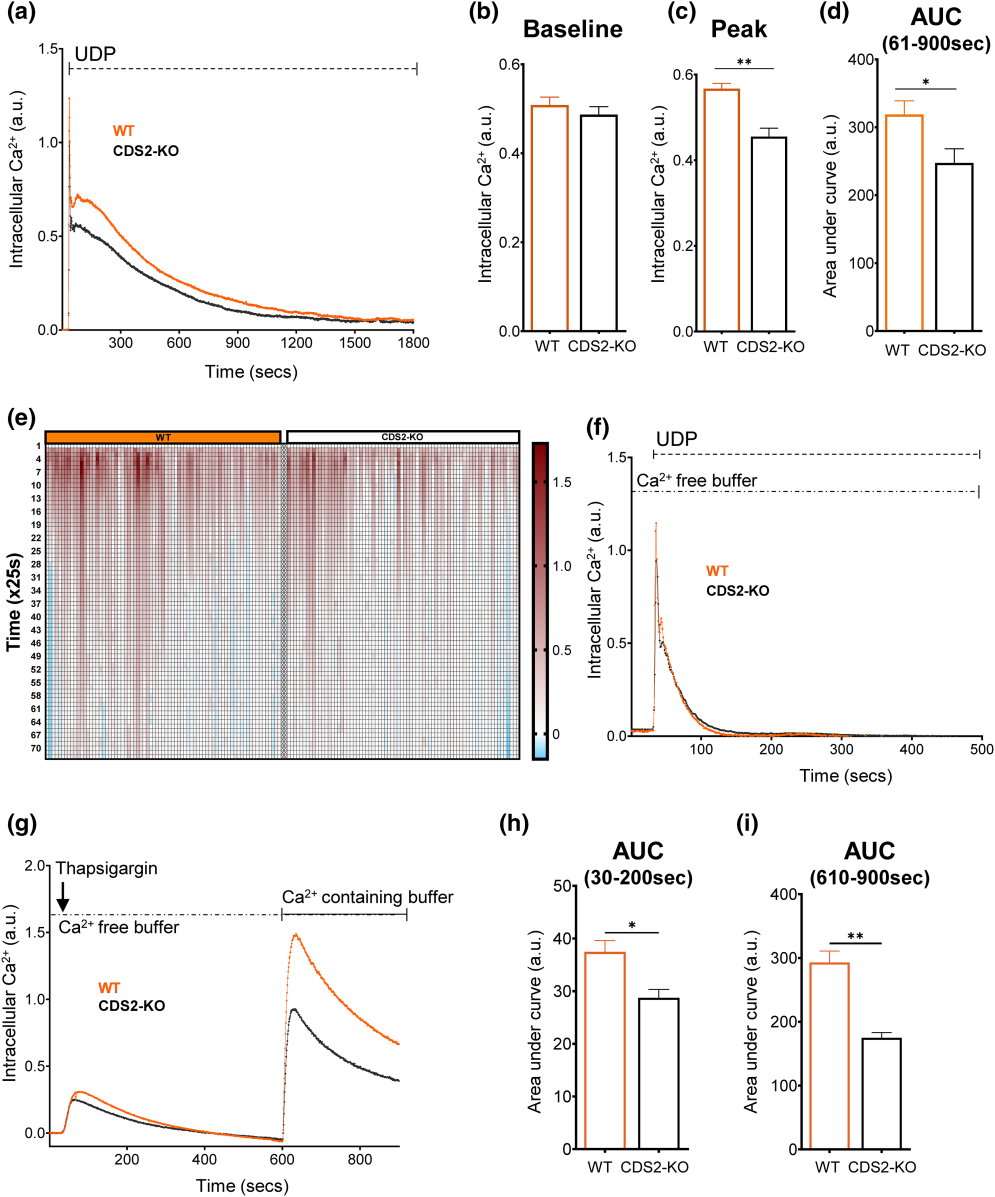
The effect of loss of CDS2 on a UDP-stimulated Ca^2+^ response. (**a**) WT or CDS2-KO macrophages preloaded with Fura-PE3/AM were incubated in Ca^2+^-containing medium and their intracellular Ca^2+^ concentration analysed by fluorescent imaging. One hundred micromolars of UDP was added at *t *=* *30 s and incubations continued for the times indicated. Data are line traces representing the mean intracellular Ca^2+^ concentration from four independent biological experiments, after subtraction of the baseline value (average value before addition of UDP). (**b–d**) Baseline (average value pre-stimulation), peak height (maximum value after addition of UDP, within the time range of 36–50 s, baseline subtracted) and integrated Ca^2+^ responses (area under curve, between 61 and 900 s after addition of UDP, baseline subtracted) in WT or CDS2-KO BMDMs for experiments described in (**a**). Data are means ± S.E.M. (*n* = 4). Statistical significance was assessed using student's paired *t*-test (*P < 0.05, **P < 0.01, ***P < 0.001). (**e**) A representative spaghetti plot illustrating the heterogeneity in Ca^2+^responses of single cells taken from one experiment described in (**a**) (*y*-axis in time x25 s). (**f**) WT or CDS2-KO macrophages preloaded with Fura-PE3/AM were incubated in Ca^2+^-free medium and their intracellular Ca^2+^ concentration analysed by fluorescent imaging. One hundred micromolars of UDP was added at *t* = 30 s and incubations continued for the times indicated. Data are line traces representing the mean intracellular Ca^2+^ concentration from four independent biological experiments (baseline subtracted). (**g**) WT or CDS2-KO macrophages preloaded with Fura-PE3/AM were incubated in Ca^2+^-free medium before addition of 2.5 µM Thapsigargin at *t* = 30 s. Incubations were continued until *t *= 600 s, at which point the medium was replaced by a wash-in of Ca^2+^-containing medium (in the absence of Thapsigargin). Data are line traces representing the mean of three independent biological experiments (baseline subtracted). (**h,i**) Area-under-curve values for the indicated time frames from experiments described in (**g**). Data are means ± S.E.M. (*n* = 3). Statistical significance was assessed using student's paired *t*-test (*P < 0.05, **P < 0.01, ***P < 0.001).

We further investigated the effect of CDS2-deletion on PLC signalling in macrophages by measuring cytosolic Ca^2+^ responses to UDP, using the fluorescent Ca^2+^-indicator Fura-2. In normal Ca^2+^-containing medium, UDP stimulated the expected biphasic Ca^2+^ response, an initial rapid spike in intracellular Ca^2+^ (previously shown to be caused by InsP_3_-mediated emptying of ER-Ca^2+^-stores), followed by a second, more-sustained phase (previously shown to be fuelled by Ca^2+^-entry from the outside, via STIM/Orai-mediated store-operated-calcium-entry) (Figure 10, Supplementary Figure S7a) [25]. Compared with the WT, CDS2-KO macrophages exhibited a slightly reduced basal cytosolic Ca^2+^ level and significantly reduced peak and integrated Ca^2+^ responses to UDP (Figure 10a–e, Supplementary Figure S7a).

We then measured Ca^2+^-responses to UDP in the absence of extracellular Ca^2+^, isolating the InsP_3_-sensitive Ca^2+^-response from store re-filling. Under these conditions, Ca^2+^ responses in WT and CDS2-KO cells were much more similar (Figure 10f), suggesting the main difference in CDS2-KO cells may be related to Ca^2+^ entry. We tested this directly by employing an experimental protocol designed to look specifically at store-operated-calcium-entry (SOCE); thapsigargin is first added to cells in Ca^2+^-free medium, leading to a steady emptying of ER Ca^2+^-pools (thapsigargin inhibits the ER Ca^2+^-ATPase), followed by the addition of extracellular Ca^2+^, to follow stimulated re-entry (Figure 10g). This protocol revealed a clear reduction in the cytosolic Ca^2+^ rise after addition of extracellular Ca^2+^ in CDS2-KO cells (Figure 10g,i). This indicates both a defect in the pathways which handle Ca^2+^ in CDS2-KO macrophages and that this defect is independent of acute stimulation of GPCRs (because it is apparent in the absence of UDP). Furthermore, the absence of any clear effect of CDS2-deletion on the initial InsP_3_-mediated Ca^2+^ response to UDP is consistent with both the lack of effect of CDS2-deletion on PIP2-levels Figure 9d) and on UDP-stimulated InsP3 formation (Supplementary Figure S7b).

## Discussion

Loss of CDS2 in primary mouse macrophages caused a significant increase in basal PA, DG, and TG. Very similar observations have now been made in several different contexts where deletion of CDS1 and/or 2 have been investigated [1,13,19–21], suggesting this phenotype is linked to a fundamental property of the way the underlying lipid biosynthetic pathways react to a loss in CDP-DG synthetic capacity. Surprisingly, however, CDS2 deletion in mouse macrophages did not cause the expected decrease in the levels of its immediate product CDP-DG, nor the predicted decrease in PI. Previous work has not measured the consequences of CDS2 deletion on CDP-DG, but the effects on PI levels have been highly variable, with reports of both minimal effects on PI [21], similar to our observations here, or substantial decreases [13,20]. This suggested to us that changes in basal lipid levels upon loss of CDS2 could not simply be explained through a ‘back-up’ in its substrate PA, which would be predicted to simply reduce the levels of its direct and indirect products, CDP-DG and PI.

To examine the effects of CDS2-deletion on the rates of the relevant biosynthetic pathways, we employed several isotopologue tracing strategies. The use of labelled glucose revealed a substantial increase in the rates of *de novo* synthesis of PA 32:0 and PA 34:1 in CDS2-KO macrophages — molecular species of PA that are characteristic of the *de novo* synthesis pathway [10]. Furthermore, they revealed minimal impact of CDS2-deletion on the *de novo* synthesis of PI 34:1, or the main product of acyl chain re-modelling of PI species made *de novo* in these cells, PI 38:4 [10].

We also analysed the total rates of phosphate incorporation into PA and PI species using an isotopologue of water. Labelled phosphate can enter the PA and PI pools via the *de novo* synthesis pathway (through glycolysis) or via the PI-cycle/recycling pathway (through direct phosphorylation of DG by DGK). This revealed a very substantial increase in the labelling of PA 38:4 and PA 34:1 in CDS2-KO macrophages. The increase in labelling of PA 38:4 indicates a substantial increase in DGK activity in CDS2-KO cells (because this is not a molecular species that is made in large quantities *de novo*). These studies also indicated very similar rates of total PI synthesis (for both PI 38:4 and PI 34:1) between WT and CDS2-KO cells, which was also confirmed by measuring the incorporation of labelled inositol.

The most parsimonious interpretation of our results is that loss of CDS2 results in a substantial increase in the *de novo* synthesis of shorter chain/more saturated species of PA, which is the likely cause of the increase in the steady-state levels of these species. This increased PA is then sufficient to support ‘normal’ levels of *de novo* CDP-DG and PI synthesis via CDS1 — those PI species synthesised *de novo* are then rapidly acyl chain remodelled to PI 38:4 by pathways known to be highly active in these cells [10], supporting the levels of the major pools of phosphoinositides.

**Figure 11.**
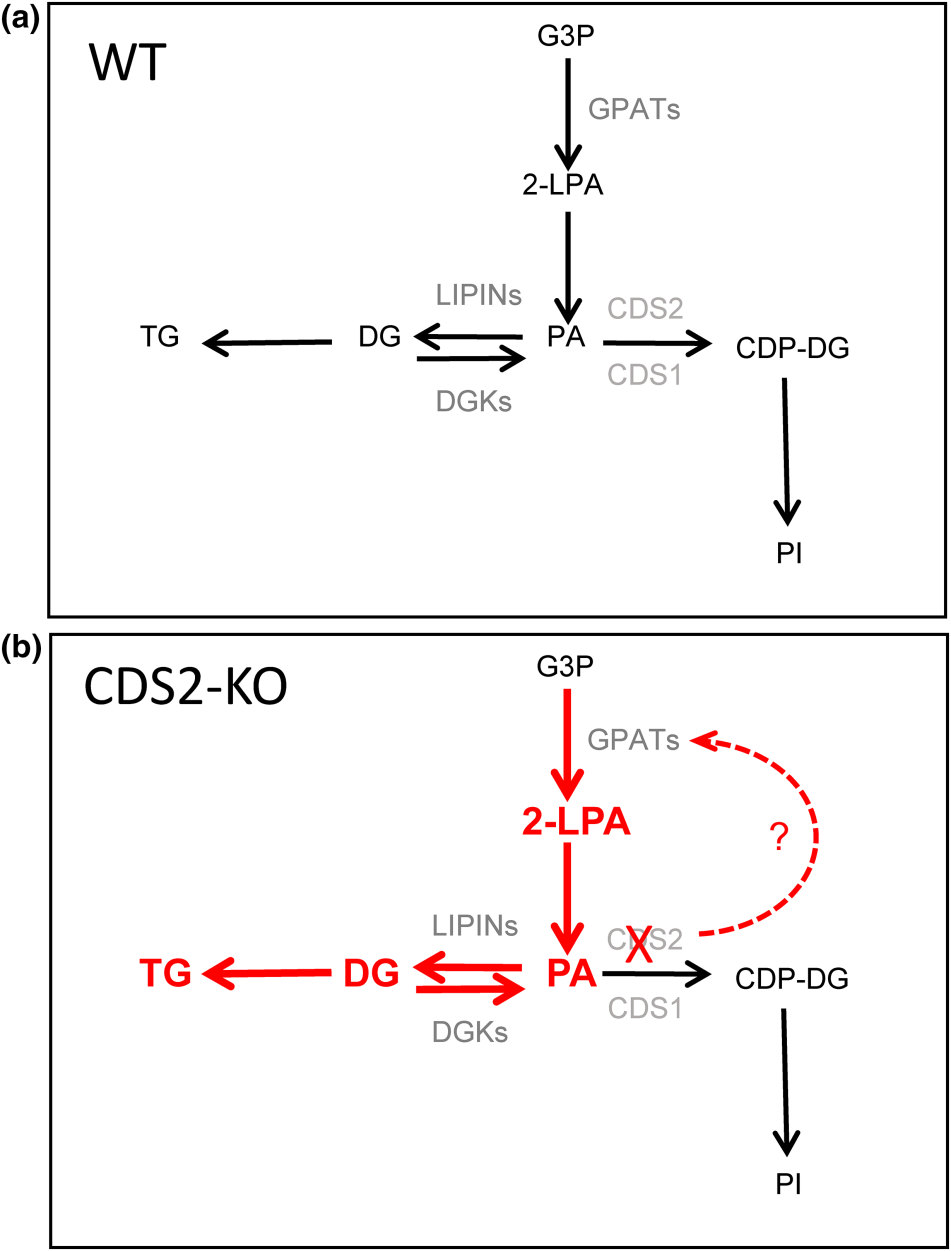
The effect of CDS2 deletion on basal lipid metabolism. (**a,b**) illustrate the effect of CDS2-deletion on relevant elements of basal lipid metabolism (see Figure 1a for the wider context). Loss of CDS2 is suggested to increase GPAT activity by an unknown mechanism, leading to increased flux through the *de novo* synthesis pathways marked in red. The increased levels of PA in CDS2-KO macrophages drives increased CDS1 activity, compensating for loss of CDS2 and maintaining basal levels of PI. This increased synthesis of PA also drives increased flux through LIPINs, leading to increased synthesis of DG and TG.

The increased pools of PA in CDS2-KO macrophages turn over with similar or increased fractional rates, indicating they are served by both increased supply and consumption. Previous work has suggested increased levels of PA in the ER are effectively channelled into the main lipid storage molecule of the cell, TG, via LIPINs and DG acyl transferases [6–8]. Thus, we consider it highly plausible that increased *de novo* synthesis of PA in CDS2-KO macrophages drives both increased flux through CDS1 (to maintain *de novo* CDP-DG/PI synthesis) and increased flux through LIPINs (to support increased synthesis of TG), see Figure 11.

Further work is needed to fully explain the changes in both size and composition of the DG and PA pools in CDS2-KO cells. PA and DG are central metabolic intermediates, with multiple routes of consumption and synthesis, making a complete diagnosis extremely challenging. Looking across the acyl chain composition of the major lipid pools, and noting the substantial increase in ^18^O-water labelling of PA 38:4, there is an argument that additional ‘38:4-backbones’ are supplied to the DG/PA/TG metabolic axis in CDS2-KO cells. The origin of these backbones is unknown and could arise at many different levels, from the supply of acyl-CoA intermediates to their selective incorporation into specific intermediates. Given our recent description of the role of 38:4-backbones in maintaining an efficient PI-cycle, and the role of CDS2 within it [10], it is tempting to speculate that CDS2-KO macrophages have a less ‘closed’ PI cycle (through either differential ER location or, the reduced preference for PA 38:4 of CDS1 compared with CDS2 [12]), resulting in a ‘leakage’ of DG 38:4 (a backbone created initially via acyl chain remodelling of *de novo* -synthesised PIs) out of the cycle, and that fuels further metabolic pathways (e.g. ‘38:4’-enriched TG synthesis). However, the total rates of PI synthesis (and hence the PI cycle) are very similar between WT and CDS2-KO cells and thus this leakage would have to be relatively small (although perhaps more clearly revealed if 38:4-enrichment of TG is measured under conditions of UDP-stimulation), or compensated by a pathway we have not measured.

It is also interesting to note that increased *de novo* synthesis of PA in CDS2-KO macrophages did not lead to increased synthesis of PG, suggesting Tam41 is relatively insensitive to increased *de novo* synthesis of PA in the ER. It would be interesting to extend these observations by tracing the rate of incorporation of labelled glucose into other major phospholipid classes to investigate the extent to which the increased *de novo* synthesis of PA observed here is channelled specifically towards TG (as might be predicted from the lack of effect on the steady state levels of the other major lipid classes).

Our data indicates the key step in *de novo* PA synthesis that is stimulated in the absence of CDS2 is the initial acylation of G3P, derived from DHAP, by GPAT acyl transferases. This aligns with previous work indicating DHAP is the triose phosphate generated by glycolysis that supplies most of the G3P used for PA synthesis [26,27]. It also aligns with previous work indicating that the primary regulated/rate limiting step in *de novo* PA synthesis is the acylation of G3P, the first committed step in this pathway [28,29]. This then immediately raises the question of how GPAT activity is increased in the absence of CDS2? We did not observe an increase in expression of GPAT3/4 in CDS2-KO macrophages, the two isoforms most clearly linked to *de novo* PA synthesis in the ER [29], suggesting a mechanism involving post-translational modification. The nature of the initial sensor that triggers these effects is also unclear. It is tempting to speculate that a small reduction in PI synthesis triggers a homeostatic feedback mechanism that operates to maintain PI levels, but such a mechanism would need to be either very sensitive, or modified by further adaptations, to be compatible with the ‘normal’ levels of PI we observe in CDS2-KO cells. It is also possible that loss of the CDS2 protein itself, rather than its catalytic activity, is the cause of the phenotype we observe, and the recent discovery of complexes containing AGPAT2/CDSs, Seipin-AGPAT2 and Seipin-GPATs, suggests that the organisation and channelling of lipid synthesis may indeed be disrupted by loss of CDS2 protein [29,30]. The elucidation of this or other potential mechanisms must now be an important goal.

Although mouse macrophages could maintain normal rates of basal PI synthesis in the absence of CDS2, they were unable to support a normal stimulated PI-cycle, resulting in a substantial decline in both PI and PIP after only 15 min stimulation with UDP. Further analysis confirmed a major bottleneck in CDS2-KO cells is the conversion of PA to PI during stimulation, leading to a rate of PI resynthesis that could not keep pace with elevated PI consumption (to maintain PIP2-directed PLC activity). Several studies have now suggested CDS2 may be preferentially suited to a role in a stimulated PI-cycle compared with CDS1 (CDS2 may have a higher selectivity, higher Vmax and a higher Km for PA 38:4, allowing it to more effectively utilise a PLC-derived increase in this species), but this preference is not absolute [1,10,12] and the phenotype we observed here could simply be due to a decrease in overall CDS capacity in the CDS2-KO cells.

We also observed a clear ‘Ca^2+^-phenotype’ in CDS2-KO cells. Loss of CDS2 most clearly reduced the rise in Ca^2+^ entry stimulated by the emptying of ER- Ca^2+^ pools (SOCE); this did not depend on the presence of UDP and both the stimulated production of InsP_3_ and the initial InsP_3_-stimulated Ca^2+^ release from ER pools appeared to be unaffected. The precise mechanism behind this effect is unclear but could be related to PA-dependant effects on ER-PM [31] or ER-mitochondrial calcium fluxes (e.g. through a PA-seipin interaction) [32,33]. Alternatively, there may be a contribution from a direct effect of PI4P on SOCE [34,35], though this would require invoking a Ca^2+^-induced PLC activity in the presence of thapsigargin leading to a similar CDS2-dependent decrease in PI4P to that seen in the presence of UDP.

CDS2 has attracted increasing attention recently as a potential target for anti-angiogenic treatment of tumours [36,37] and for treatment of uveal melanomas driven by mutationally enhanced PLC signalling [38]. Deletion of CDS2 has also been used recently to create a mouse model of hepatic steatosis [21]. Our data support an important role for CDS2 in supporting a highly stimulated PI-cycle and begin to unpick a wider phenotype caused by loss of CDS2 activity that will need to be understood before CDS2 can be considered a rational therapeutic target.

## Materials and methods

### Materials

[^13^C_6_, 99%]-glucose was from Sigma–Aldrich. [^13^C_6_D_7_, 97–98%]-glucose and [^18^O, 97%]-water were from Cambridge Isotopes. [^18^O/^2^H]- inositol was synthesised as described previously [10].

All chemicals/solvents for lipid analysis were AR grade. M-CSF was from PeproTech, Uridine 5-diphosphate (UDP), myo-inositol, D-Glucose, HEPES and 2X DMEM were from Sigma. L-Glutamine, heat-inactivated foetal bovine serum, dialysed foetal bovine serum, Penicillin–Streptomycin, Trypsin–EDTA (0.05%), DMEM/F12, and DMEM without glucose were from Gibco. Inositol-free DMEM/F12 was from BioConcept.

PI, LPI, DG, PG, PA, PC, PE, PS, CL and TG internal standards used for lipidomic analyses by Orbitrap LC–MS (Figure 2) were purchased from Avanti and used as previously detailed [39]. Internal standards used in Triple Quad LC–MS/MS lipidomic analysis were as follows: LPA 17:1 was from Avanti; d6-PI4P 18:0/20:4, d6-DG 18:0/20:4, PA 17:0/16:0, PI 17:0/16:0 and PI(3,4,5)P3 17:0/16:0 (as hepta-sodium salts), were synthesized at the Babraham Institute.

### Generation of mice with conditional deletion of CDS2 in the myeloid lineage

Mice were generated using CDS2-targeted embryonic stem cells [KOMP ES cell line *Cds2^tm1a(KOMP)Wtsi^*, RRID:MMRRC_053527-UCD, was obtained from the Mutant Mouse Resource and Research Center (MMRRC) at University of California at Davis, an NIH-funded strain repository, and was donated to the MMRRC by The KOMP Repository, University of California, Davis; Originating from Pieter de Jong, Kent Lloyd, William Skarnes, Allan Bradley, Wellcome Sanger Institute]; see Figure 1. The promoter-less LacZ/neo selection cassette prevents expression of CDS2 and no live births were recorded for mice homozygous for this allele, indicating that the KO of CDS2 is embryonic lethal:

**Table:** 

	CDS2+/+ (WT)	CDS2+/− (Het)	CDS2−/− (KO)
No. of live births	46	79	0
% of total	36.8	63.2	0

The LacZ/neo selection cassette in heterozygotes was then excised through an initial cross with mice expressing FLPe (CAG-FLPe deleter mice [40]). The resulting mice with loxP-flanked exon-2 of CDS2 (CDS2^loxP^) were then crossed with mice expressing LysMCre (Lyz2^tm1(cre)Ifo^; Jackson Laboratory) to generate mice with myeloid deletion of exon-2 of CDS2. The colony was maintained by interbreeding mice both homozygous for LysMCre and heterozygous for ‘floxed’ CDS2 (CDS2^wt^/^loxP^) to generate offspring that were homozygous for LysMCre and either homozygous for WT CDS2 (‘WT’), homozygous for ‘floxed’ CDS2 (CDS2^loxP/loxP^; ‘CDS2-KO’) or, heterozygous for ‘floxed’ CDS2 (CDS2^wt^/^loxP^).

Routine genotyping of pups for CDS2-targetted exon-2 was performed by PCR using:

‘Forward primer’ — TCTCAGGCTTCGTTTCG’; ‘Reverse primer’ — CCTTTCCAACCTGTCCT

WT and CDS2KO mice used in all experiments were on a final mixed C57BL/6J; C57BL/6; C57BL/6Babr background.

All mice were housed in the Biological Support Unit at the Babraham Institute under specific pathogen–free conditions. All animal experiments at The Babraham Institute were reviewed and approved by The Animal Welfare and Ethics Review Body and performed under Home Office Project license PPL 70/8100. Mice were humanely sacrificed using schedule 1 methods (by CO_2_ inhalation and death confirmed by cervical dislocation).

### Culture of BMDM

BMDM were prepared from WT and CDS2-KO mice of 8–20 weeks of age, as previously described [41] and cultured at 37°C and 5% CO_2_ in DMEM/F12 supplemented with 10% foetal bovine serum, M-CSF (10 ng/ml day 1-3; 20 ng/ml day 3–6) and 1% w/v penicillin/ streptomycin. Cells were routinely seeded at day 6 post-dissection into TC-treated dishes medium containing 10 ng/ml M-CSF, then incubated overnight to achieve ∼80% confluence at the time of the experiment. During the course of this study, we noticed that CDS2-KO cells generally grew slightly slower than WT cells, and thus typically we seeded ∼25% fewer WT cells on day 6 to achieve comparable cell numbers on experimental day 7 (12-well at 1.8 × 10^5^–2.4 × 10^5^/well; 6-well at 4 × 10^5^–5 × 10^5^/well; 10 cm dish at 2.5 × 10^6^–3 × 10^6^/well).

### Lipidomic analyses by Orbitrap LC–MS

Cells were washed in ice-cold 1xPBS twice and then collected by scraping and pipetting into a pre-chilled 2 ml polypropylene Eppendorf tube (typically, cells in a 10 cm dish were collected in a total volume of 1.2 ml ice-cold 1xPBS). Cell debris was then centrifuged for 15 s at 15 000***g***/4°C, the supernatant aspirated and the pellet immediately snap frozen in liquid N_2_. Pellets were stored at −80°C until analysis by MS.

For the broad analysis of phospholipids, neutral lipids, cholesterols and sphingomyelin, a Folch solvent extraction was performed, followed by LC–MS using an Orbitrap Elite spectrometer (Thermo Fisher), as described in [39]. Response ratios were calculated for the peak areas of endogenous species divided by relevant internal standards and normalisation between biological replicates was performed using DNA quantification (using 2 µl of the Folch upper phase and a Nanodrop reader — Thermo Fisher Scientific, NanoDrop™2000/2000c).

### Lipidomic analyses by Triple Quad LC–MS/MS

For the targeted analysis of PA, PI and PIP2: cell incubations were terminated by aspiration of the medium, followed by the addition of ice-cold IM HCL (typically cells in six well dishes were scraped and collected in a total volume of 1 ml ice-cold HCL in 2 ml polypropylene Eppendorf tubes). Cell debris was then centrifuged for 10 min at 15 000***g***/4°C, the supernatant aspirated and the pellet snap frozen in liquid N_2_. Lipids were extracted using an acidified Folch phase partition, derivatised with TMS-diazomethane and analysed by LC–MS/MS using a Triple Quadrupole mass spectrometer (ABSciex QTRAP), as described previously [10,42].

For analysis of LPA, cells were seeded in a 10 cm dish at 3 × 10^6^–4 × 10^6^/well. Cell incubations were terminated with ice-cold methanol (1 ml/dish) and collected into 2 ml pre-chilled 2 ml polypropylene Eppendorf tubes. Cell debris was centrifuged for 10 min at 15 000***g***/4°C and the supernatant transferred into MS vials and immediately dried down under a steady stream of N_2_ gas. The samples were then resuspended with 50 µl of 80% MS grade methanol followed by gentle vortex before analysis by LC–MS/MS using the following conditions and parameters. Five microlitres of sample mix was injected onto a Kinetex EVO C18, 2.6 µm, 100 Å, 100 × 2.1 mm column. HPLC conditions were:

Solvent A: 5 mM ammonium formate in water + 0.5% formic acid
Solvent B: 5 mM ammonium formate in 5% water + 95% acetonitrile + 0.5% formic acid

HPLC gradient:

**Table:** 

Time/min	Flow rate ml/min	% solvent B
0	0.2	10
8	0.2	10
13	0.2	50
25	0.2	95
37	0.2	95
37.1	0.2	10
40	0.2	10

Mass spectrometer parameters were:

Sciex QTRAP 6500 in low mass mode.

Polarity: Negative.

Mass spectrometer method Parameter Table

CUR: 20.00
TEM: 400.00
GS1: 40.00
GS2: 30.00
CAD: −2.00
IS: −4500.00
DP: −200.00
EP: −10.00
CE: −30.00
CXP: −13.00

Mass table for LPA analysis

**Table:** 

Q1 mass (Da)	Q3 mass (Da)	Dwell (ms)	ID
409.236	153	40	LPA 16:0
412.236	156	40	LPA 16:0 13C3
407.22	153	40	LPA 16:1
410.22	156	40	LPA 16:1 13C3
423.252	153	40	LPA 17:0 (ISD)
437.267	153	40	LPA 18:0
440.267	156	40	LPA 18:0 13C3
435.252	153	40	LPA 18:1
438.252	156	40	LPA 18:1 13C3
433.236	153	40	LPA 18:2
436.236	156	40	LPA 18:2 13C3
431.22	153	40	LPA 18:3
434.22	156	40	LPA 18:3 13C3
461.267	153	40	LPA 20:2
464.267	156	40	LPA 20:2 13C3
459.252	153	40	LPA 20:3
462.252	156	40	LPA 20:3 13C3
457.236	153	40	LPA 20:4
460.236	156	40	LPA 20:4 13C3

For the targeted analysis of CDP-DG: cells were washed and collected in ice-cold 1xPBS, as described above (typically, cells were harvested from 10 cm dishes). Cell pellets were snap frozen in liquid N_2_ and stored at −80°C. Pellets were then extracted with methanol/tert-Butyl methyl ether/water, the upper ether layer discarded, and the lower phase extracted with acidified chloroform/methanol/water, followed by derivatisation with TMS-diazomethane and analysis by LC–MS/MS, as described in [10].

For the targeted analysis of DG: cells were washed in ice-cold PBS, and scrapped into ice-cold methanol (typically, cells in six-well dishes were collected into a total volume of 300 μl methanol in pre-chilled 2 ml polypropylene Eppendorf tubes). A neutral chloroform/methanol/water lipid extraction was then performed immediately, and DG species analysed by LC–MS/MS, as described in [10].

Response ratios were calculated for the endogenous species divided by the relevant internal standards (except for CDP-DG, where an appropriate internal standard was not available) and normalisation between biological replicates was performed as described in the figure legends (typically to either cell number, or total PC measured in the same lipid extracts).

### Stable labelling of lipids

All media for glucose (Glc) isotopologue labelling experiments were prepared by making a master solution of Glc-free DMEM (Gibco 11966-025) supplemented with 10% dialysed FBS (Gibco, A3382001), 20 mM 4-(2-hydroxyethyl)-1-piperazineethanesulfonic acid (HEPES) (Gibco, 15630-056) and 2 mM L-Glutamine. The appropriate volumes of master medium were then aliquoted into separate tubes and 10 mM final concentration of either [^12^C_6_]-Glc (stock 1 M; Thermo Fisher Scientific, G/0500/53) or, [^13^C_6_]-Glc (stock 2.5 M; Sigma–Aldrich, 389374) or, [^13^C_6_D_7_]-Glc (stock 2.5 M; CK isotopes, CDLM-3813-1) was added. Stocks were stored in −20°C.

Cells were routinely seeded at a density of 1.8 × 10^5^/dish and 2.4 × 10^5^/dish for WT and CDS2-KO cells, respectively, for PI and PA labelling and 3 × 10^6^/dish and 4 × 10^6^/dish for WT and CDS2-KO, respectively, for LPA labelling and incubated in complete medium with 10 ng/ml M-CSF overnight. Media was then aspirated and replaced with the appropriate pre-warmed labelling media (250–300 µl/per 12 well and 5 ml/per 10 cm dish) for the times indicated. Cell incubations were routinely quenched with 1 M ice-cold HCl, and lipids analysed as described above for PA and PI (with masses adjusted for the inclusion of additional isotopes). For LPA labelling, cell incubations were terminated with ice-cold methanol (1 ml/dish) and collected into 2 ml pre-chilled 2 ml polypropylene Eppendorf tubes.

For [^18^O]-H_2_O labelling, a master medium was made with 2xDMEM medium (Millipore, SLM-202-B) supplemented with 20% dialysed FBS, 20 mM HEPES solution and 4 mM L-Glutamine. The volume of the master medium was split into two equal parts to which either [^18^O]-H_2_O (CK isotopes, Water ^18^O, 97%, OLM-240) or MilliQ H_2_O was added at a 1:1 ratio. Routine [^18^O]-H_2_O labelling was performed in a similar manner to glucose labelling described above.

### Analysis of lipid droplets

BMDMs were seeded in six-well TC treated plates containing sterile 14 mm round glass coverslips at a density of 3.6 × 10^5^ cells/well for WT BMDMs and 4 × 10^5^/well for CDS2-KO BMDMs in complete medium and 10 ng/ml M-CSF. The cells were incubated overnight at 37°C/5% CO_2_ after which they were washed with 1xPBS solution, followed by the addition of 500 µl/well of 4% (w/v) paraformaldehyde (Sigma–Aldrich, F8775) for 10 min at RT to fix the cells. Wells were washed three times with 1xPBS to remove paraformaldehyde. One millilitre of lipid droplet stain, 4,4-difluoro-4-bora-3a,4a-diaza-s-indacene (BODIPY 493/503 nm)-PBS solution was added to each well (at working concentration of 10 µg/ml prepared from 2 mg/ml stock solution stored at −20°C, Cayman Chemicals, 121207-31-6). The fixed cells were then incubated for 15 min at RT in the dark to minimise photobleaching of the stain, then washed three times with 1xPBS. Coverslips containing the fixed and stained cells were mounted onto microscope slides with 2-(4-amidinophenyl)-1H -indole-6-carboxamidine (DAPI) containing mounting medium (Invitrogen Prolong Diamond Antifade Mountant with DAPI, P36966) and left in the dark at RT overnight before imaging. Coverslips were imaged using a Nikon A1R confocal microscope at a magnification of 60×. Images were stored as .nd files and lipid droplet puncta analysed using the Imaris software system. Image and scale bar presentation were analysed using the ImageJ software system.

### Analysis of inositol phosphates by CE-ESI-MS

BMDMs were seeded in 10 cm TC treated plates at a density of 3 × 10^6^ cells/plate and incubated overnight in complete medium and 10 ng/ml M-CSF. Prior to stimulation, medium was aspirated from the plates and replaced with 5 ml Ca^2+^-containing buffer prewarmed to 37°C (155 mM NaCl; 5 mM KCl; 2 mM NaH_2_PO_4_; 1 mM MgCl_2_; 2 mM CaCl_2_; 10 mM HEPES; 10 mM Glucose; pH 7.2). Where indicated, cells were stimulated with 100 µM UDP in Ca^2+^-containing buffer before termination by the addition of 1.4 ml/plate 1 M perchloric acid (Sigma–Aldrich, 24425-2). Cells were left to incubate for 15 min on ice before scrapping and collection into 2 ml Eppendorf tubes on ice. Cells were pelleted by centrifugation at 18 000***g*** for 5 min at 4°C and the supernatant transferred into fresh 2 ml ice-cold Eppendorf tubes containing 4 mg of titanium dioxide beads (TiO, GL Sciences Inc., 5020-75000; pre-equilibrated in MilliQ H_2_O). Inositol phosphate purification on the TiO_2_ beads and analysis of inositol phosphates by CE-ESI-MS was as described previously [43,44].

### Measurement of intracellular Ca^2+^

WT and CDS2-KO BMDMs were seeded at a density of 3.5 × 10^5^ cells/well into six-well TC treated plates containing 22 mm diameter #1.5 glass circular coverslips (VWR International) and incubated overnight in complete medium plus 10 ng/ml M-CSF. Prior to imaging, cells were washed with Ca^2+^-containing buffer and then incubated with 1 ml/well Ca^2+^-containing buffer + 1 mM Fura-PE3-AM preheated to 37°C [Fura-PE3-AM (1 mM in DMSO; Sigma–Aldrich, 344911) and pluronic acid (20% in DMSO; Sigma–Aldrich, P2443) were mixed 1:1 and then added to Ca^2+^-containing buffer to a final concentration of 1 µM Fura-PE3]; the cells were then left for 30 min in the dark at RT. Cells were then washed once and incubated in Ca^2+^-containing buffer (2 ml/well) for a further 30 min in the dark at RT. The buffer was then aspirated and replaced with fresh Ca^2+^-containing buffer before imaging.

Coverslips were mounted into custom rings comprising a stainless-steel upper section and a Teflon lower section. The coverslip/ring assembly formed a small chamber into which buffer could be added/exchanged, with the majority of the coverslip surface area accessible to imaging from below using an inverted microscope. Live cell imaging was performed at 37°C using an Olympus CellR widefield microscope system comprising Olympus BX41 frame, Olympus UApo/340 20x/0.75 objective, Hamamatsu Orca ER CCD camera, TILL Polychrome V monochromator light source, Solent Scientific full-enclosure incubator. The system was controlled using Olympus xCellence software. Pairs of Fura-PE3 images were captured every second using excitation at 340/7 nm and 380/7 nm, with the emitted light passing through a 525/40 nm bandpass filter (Semrock). All images were acquired using a 50 ms exposure time and a 4 × 4 bin (pixel size 1.29 mm).

Images were recorded for 30 s before any addition buffer exchange to gain a baseline reading. Where indicated, UDP was added to a final concentration of 100 µM. In some experiments, cells were prewashed and incubated with Ca^2+^-free buffer before imaging. Additionally, for experiments involving the addition of thapsigargin (TG; Invitrogen, T7458), an aspiration system was set up to maintain the incubation volume as TG was gently perfused into the chamber using a 5 ml syringe (2.5 µM final concentration in Ca^2+^ free buffer).

Images were analysed using ImageJ/FIJI with regions of interest (ROIs) manually selected and measured using the ROI manager function for each wavelength, including a reference background ROI. Background-corrected ratio values were calculated in Excel to gain a response curve. Area-under curve (AUC) analysis was computed using the trapezoidal method in Prism software. By taking the area of a typical trapezoid in the response curve, Prism computes the area of the equivalent rectangle and then calculates the sum of areas of all the rectangles presented under the curve.

### Western blots

BMDMs were seeded in six-well TC plates at a density of 5 × 10^5^ cells/well and incubated overnight in complete medium and 10 ng/ml M-CSF. The cells were then washed in ice-cold 1xPBS, and lysed directly in 120 µl/well of 1× Laemmli buffer (62.5 mM Tris base pH 6.8, 2% SDS, 10% glycerol, 5% 2-mercaptoethanol and 0.01% bromophenol blue). Lysates (6 μg, determined by Pierce™ BCA Protein Assay Kit (Thermo Fisher)) were then loaded onto an SDS–PAGE gel, without boiling (CDS2 is a highly hydrophobic protein that tends to aggregate after boiling), and Western blotted as previously described [10].

### RT-PCR

BMDMs were seeded in six-well TC plates at a density of 4 × 10^5^ cells/well and incubated overnight in complete medium and 10 ng/ml M-CSF. Cells were washed in ice-cold 1xPBS solution and then RNA extracted using QIAshredder® (Qiagen, 79654) and the RNeasy® mini kit (Qiagen, 74104). The yields of RNA extracts were determined by NanoDrop readings and were stored at −20°C. cDNA was generated from RNA extracts using the QuantiTect® RT kit (Qiagen, 205311) with the Eppendorf epgradient cycler machine, as per kit instructions. The cDNA yield was measured by NanoDrop and stored at −20°C. qRT-PCR was performed using SYBER Green Jumpstart™ Taq Ready Mix kit (Sigma–Aldrich, S4438). All steps were carried out as per the manufacturer's protocol on a standard flat-bottom 96-well plate. Run cycles on a Bio-Rad CFX96-3 included 95°C for 2 min; 94°C for 15 s; 60°C for 30 s; 72°C. for 30 s (plus plate read) for 40 cycles. Data was normalised to the housekeeping gene, hypoxanthine phosphoribosyltransferase (HPRT). Primers for the selected genes of interest were designed using the PrimerQuest tool from integrated DNA technologies and purchased from Merck. Primers were reconstituted in diethyl pyrocarbonate-treated H_2_O to the desired concentrations and stored at −20°C. Table 1 provides details of the primers used.

**Table:** 

Gene name	Primer sequence
DGKE NM_001362845 FWD	GAC GTA AAG GAA GCC GAG TAA G
DGKE NM_001362845 REV	GGT GGC TCA CAC ACA CAA TA
LIPIN1 NM_001355598 FWD Set 1	CAG GAA GGA TGC CGC TAA A
LIPIN1 NM_001355598 REV Set 1	CAG CCC AGT GAC AGT AAA GAA
LIPIN2 NM_001357791 FWD Set 1	CCA TCA CCA CCA CAG TAA GAA
LIPIN2 NM_001357791 REV Set 1	GTG AGG CAA AGA CTC CCT AAA
LIPIN3 NM_022883.3 FWD Set 2	CCC AGA GAG TAA GGA AAC CAA G
LIPIN3 NM_022883.3 REV Set 2	GGA GTA TGG CTC TCT GGA GTA
GPAT4 XM_011242104 FWD Set 1	CCA GAA GGA ACC TGC ATC AA
GPAT4 XM_011242104 REV Set 1	GCT TGA TAG CCA CAG GGT AAA
CDS1 NM_173370.3 FWD Set 2	CTT CCT CCT GTG TGT GAA CTA C
CDS1 NM_173370.3 REV Set 2	TGA ACC TGT GGT AGC GAA TG
AGPAT1 XM_006524647 FWD Set 1	TCC GAT ACA AGG GTG GAA GA
AGPAT1 XM_006524647 REV Set 1	CAA GTC CAT TGT CCA GTG TAG G
AGPAT2 NM_026212.2 FWD Set 3	CGT GGT GTA CTC GTC TTT CTC
AGPAT2 NM_026212.2 REV Set 3	GAC AGC ATC CAG CAC TTG TA
AGPAT3 NM_001404875 FWD Set 1	CCT CCT CCT ATT CTG CCT CTA T
AGPAT3 NM_001404875 REV Set 1	GTG TAT GTC ACT GAG GTG GTA AG
AGPAT5 NM_026792.3 FWD Set 5	AGC TCT AAG GCG ATG AAA GTC
AGPAT5 NM_026792.3 REV Set 5	CAG AGC CAG GGC TGA AAT AA
GPAT2 XM_006499132 FWD Set 1	CCC TCT TCC ACA GAA GCA TAA T
GPAT2 XM_006499132 REV Set 1	GGA CCA CAG GAA ATA GCA GAG
AGPAT4 XM_006523347 FWD Set 1	AGA TGT TGT CCC AGC TGT ATA TG
AGPAT4 XM_006523347 REV Set 1	CGT AGC AGT CAG CGT GAT ATT
GPAT3 XM_011249433 FWD Set 1	CTC CTC ACA CGA ACC AAT GT
GPAT3 XM_011249433 REV Set 1	CAG AGG TAG CAG GAA GCA ATA G
**CDS2ABNM_138651.7FWDEX2**	**GGG CAA GCC ACT CTC TAT TT**
**CDS2ABNM_138651.7REVEX2**	**ACA TCG TCC CTC TGT CCT AA**
**CDS2CNM_001291039FWDEX2**	**GCC TGA TTT GAG TGA TGT GAT TT**
**CDS2CNM_001291039REVEX2**	**TCT CCT CAA ACG TGC CAT AC**

### Statistical analysis

Statistical interpretation of experiments are shown for each figure within the figure legends. For statistical tests, data from n biological replicates were pooled and represented as mean ± S.E.M. Where applicable and meeting parametric assumptions, data was first log transformed prior to analysis. For broad overview of steady-state levels of major lipid species analysis log transformed paired student's *t*-test for individual species was used to analyse the changes in different molecular species. Similarly, one sample *t*-test or multiple paired *t*-test were chosen for experiments involving labelled species. Two-way ANOVA with Šídák's/Tukey's test to correct for multiple comparisons was used for experiments involving UDP-stimulation and student's paired *t*-test for UDP-stimulated Ca^2+^ imaging. AUC analysis followed the trapezoid rule computed on Prism software followed by student's paired *t*-test.

## Data Availability

The findings of this study are supported by the data within the article and its supplementary materials. Additional information can be obtained by contacting the corresponding authors.

## Abbreviations

AGPAT: acylglycerol phosphate acyltransferase
AUC: area-under curve
BMDM: bone marrow-derived macrophages
GPAT: glycerol phosphate acyltransferase
HPRT: hypoxanthine phosphoribosyltransferase
PA: phosphatidic acid
PG: phosphatidylglycerol
PGP: phosphatidylglycerol phosphate
ROI: regions of interest
SOCE: store-operated-calcium-entry
WT: wild-type
