# Supplementary material for: CDS2 expression regulates de novo phosphatidic acid synthesis
Source: Biochem J. 2024 Oct 11;481(20):1449–73. doi: 10.1042/BCJ20240456 (PMC11555650; doi:10.1042/BCJ20240456)
Supplement: Supplementary Material 1 [file BCJ-481-1449-s1.pdf]

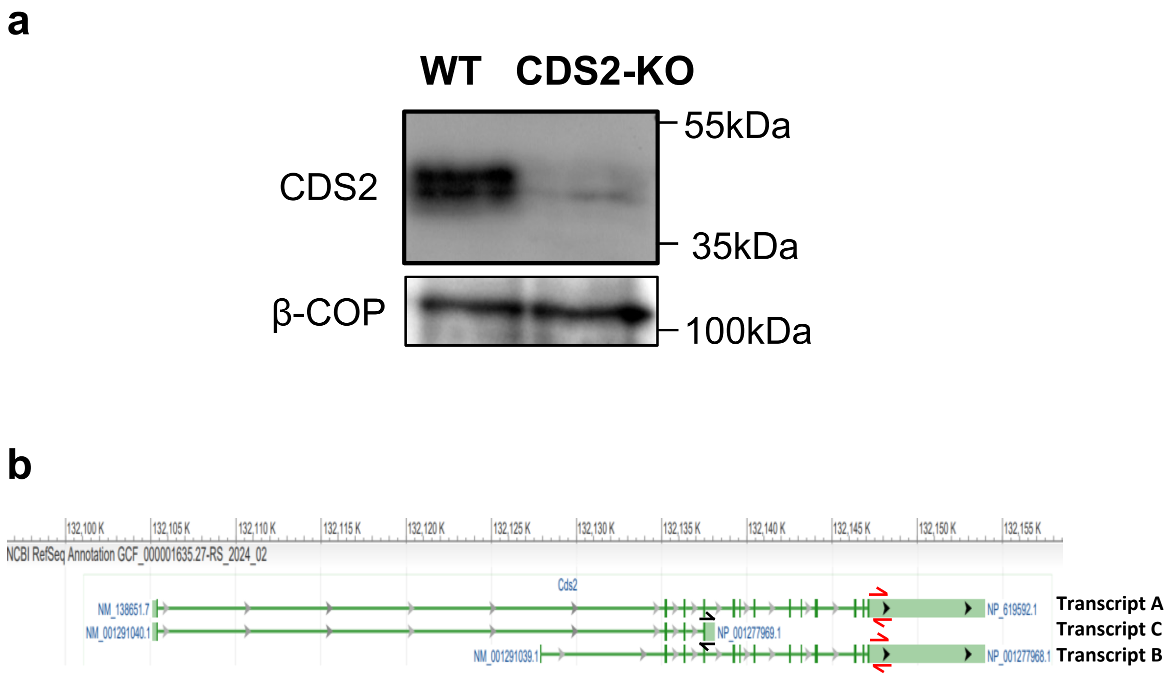

Figure S1. Validation of CDS2-KO mouse macrophages

a) Representative western blot for CDS2 in WT and CDS2-KO mouse macrophages. Cells were lysed directly in lamelli sample buffer and blotted for CDS2, and β-COP as a loading control, as described in Materials and Methods. An uncropped image of the immunoblot from which these images were derived can be found in Supplementary Figure S8.

b) Schematic from NCBI database illustrating the three potential transcripts of CDS2 (A, B and C), and position of primers used in qRT-PCR analysis of mRNA to distinguish transcripts (Primers for transcripts A and B in red, transcript C primers in black). NM_138651.7 (transcript A) represents the longest transcript variant of CDS2. NM_001291039.1 (transcript B) contains alternate splicing at the 5' terminal exon which differs in the 5' untranslated region (UTR) and 5' coding region in comparison to transcript A. The splicing event of transcript B produces a distinct N-terminus and is shorter than transcript A. NM_001291040.1 (transcript C) lacks several 3' exons and alternate splicing at the 3' terminal exon. It differs in the 3' UTR and 3' coding region in comparison to transcript A. The splicing event of transcript C produces a distinct C-terminus and is shorter than transcript A.

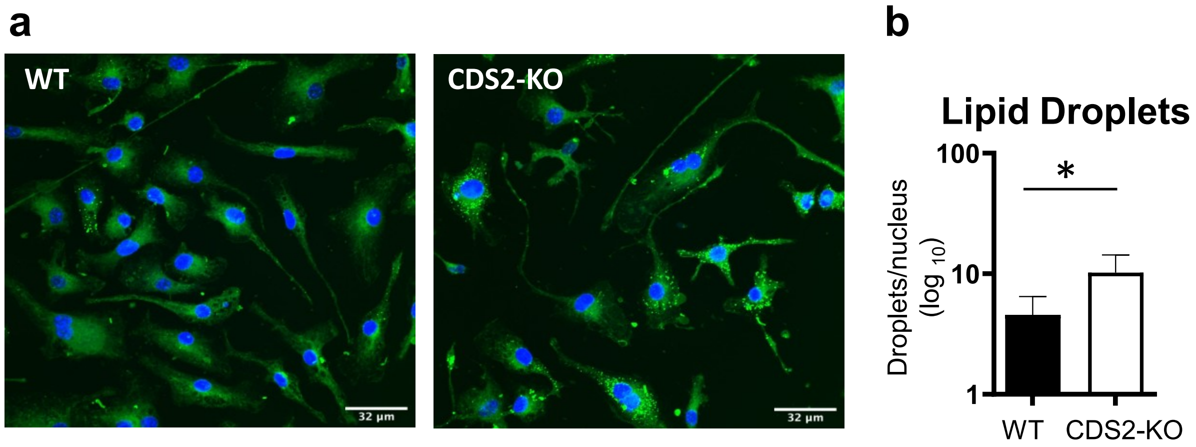

Figure S2. The effect of loss of CDS2 on lipid droplet formation in BMDMs.

a) WT or CDS2-KO BMDMs were seeded overnight onto 14 mm glass coverslips. Lipid droplets were stained with BODIPY 493/503 and imaged with Nikon A1R confocal microscopy for WT CDS2-KO BMDMs as indicated.

b) Lipid droplets were quantified using Imaris software with the data collated and represented as the number of lipid droplets per nucleus. Data are from three independent biological experiments (n=3) with a total of 448 WT and 469 CDS2-KO cells analysed. Statistical analysis was assessed using a *ratio-paired student's t-test* (*p<0.001).

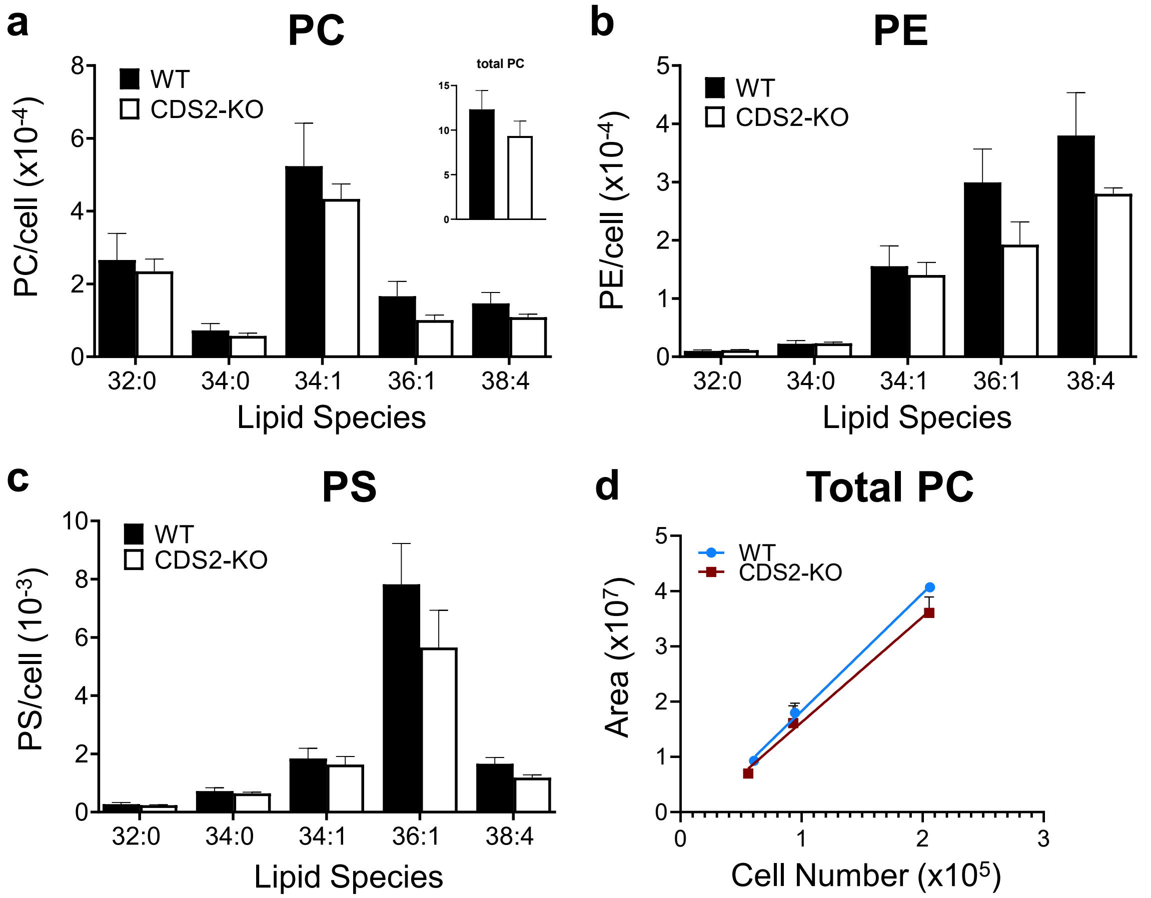

Figure S3. Focussed analysis of selected lipid species in WT and CDS2-KO macrophages. **a-c)** The indicated lipid species in WT or CDS2-KO macrophages were analysed by LC-MS/MS. Data are peak areas normalised to total cell number (quantified from parallel samples by haemocytometer). **d)** WT and CDS2-KO BMDMs were seeded at a range of cell densities. Following 24 hrs culture, 'total PC' represents the addition of the 5 x species measured as for a) and cell counts (performed in triplicate by haemocytometer) were taken in parallel samples to determine cell number per well for each genotype. Data are mean ± S.E.M of three independent biological experiments. Coloured lines represent the line of best fit. Statistical significance was assessed using a *ratio-paired student's t-test.*

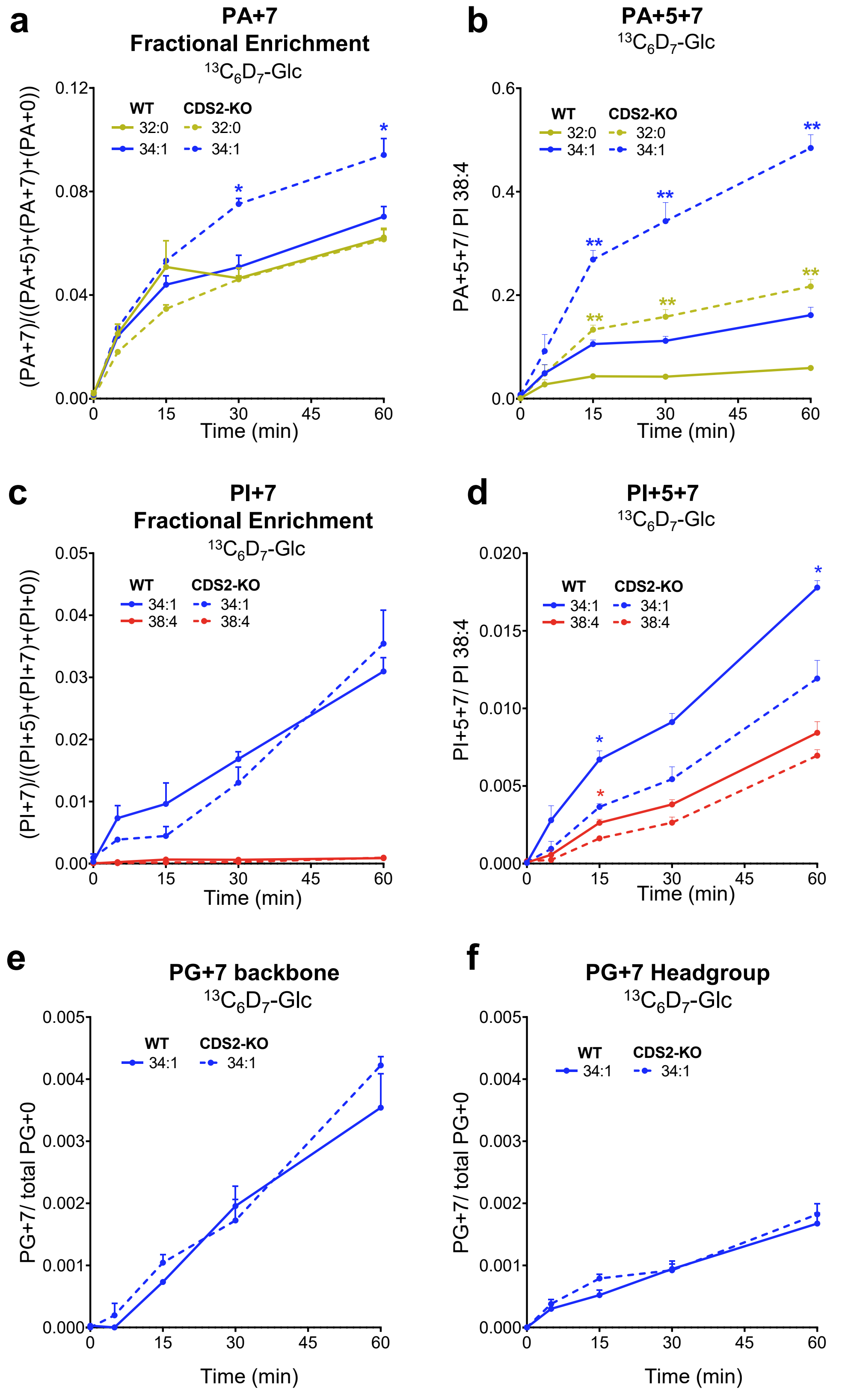

Figure S4. The rates of *de novo* synthesis of PA, PI, and PG in WT and CDS2-KO macrophages assessed by labelling with $^{13}C_6D_7$-Glucose.

$^{13}C_6D_7$-glucose was added at t=0 and the incorporation of either +5 or +7 Da into the indicated isotopologues in WT and CDS2-KO macrophages were analysed by LC-MS/MS at the times indicated. **(a-d)** Data are response ratios (peak areas corrected for the recovery of the relevant internal standards) normalised to PI 38:4 at t=0 (**b**,**d**) and represent means +/- S.E.M (n=3-4). “Fractional enrichment” is defined as the ratio of the indicated +7-isotopologue divided by the sum of labelled and unlabelled isotopologues. (**a**,**c**) Data are peak areas (no PG internal standard was available), divided by total unlabelled PG (**e**,**f**). Statistical significance was assessed using *multiple paired t-test* (*p<0.05, **p<0.01).

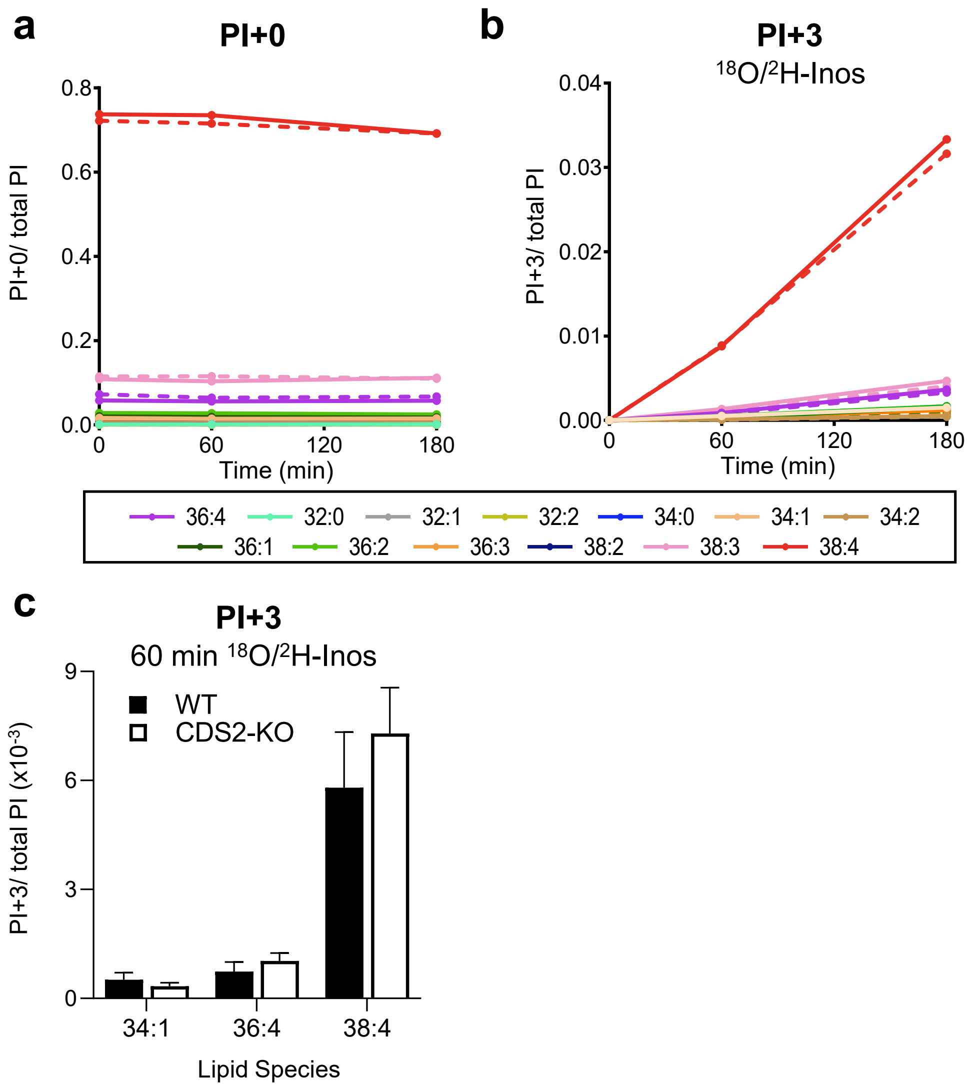

Figure S5. The rates of *de novo* PI synthesis in WT and CDS2-KO macrophages assessed by labelling with $^{18}O/^{2}H$-inositol.

$^{18}O/^{2}H$-inositol was added at t=0 and the incorporation of +3 Da in WT and CDS2-KO macrophages were analysed by LC-MS/MS at the times indicated. **(a,b).** Data are response ratios (peak areas corrected for the recovery of the PI internal standard) normalised to total PI (the addition of all the response ratios for all acyl chain species measured) and is a representative experiment of three. **c)** Data are means +/- S.E.M for PI+3 values in WT and KO BMDM at 60min $^{18}O/^{2}H$-inositol labelling (n=3). Statistical significance was assessed using a *ratio-paired student's t-test*.

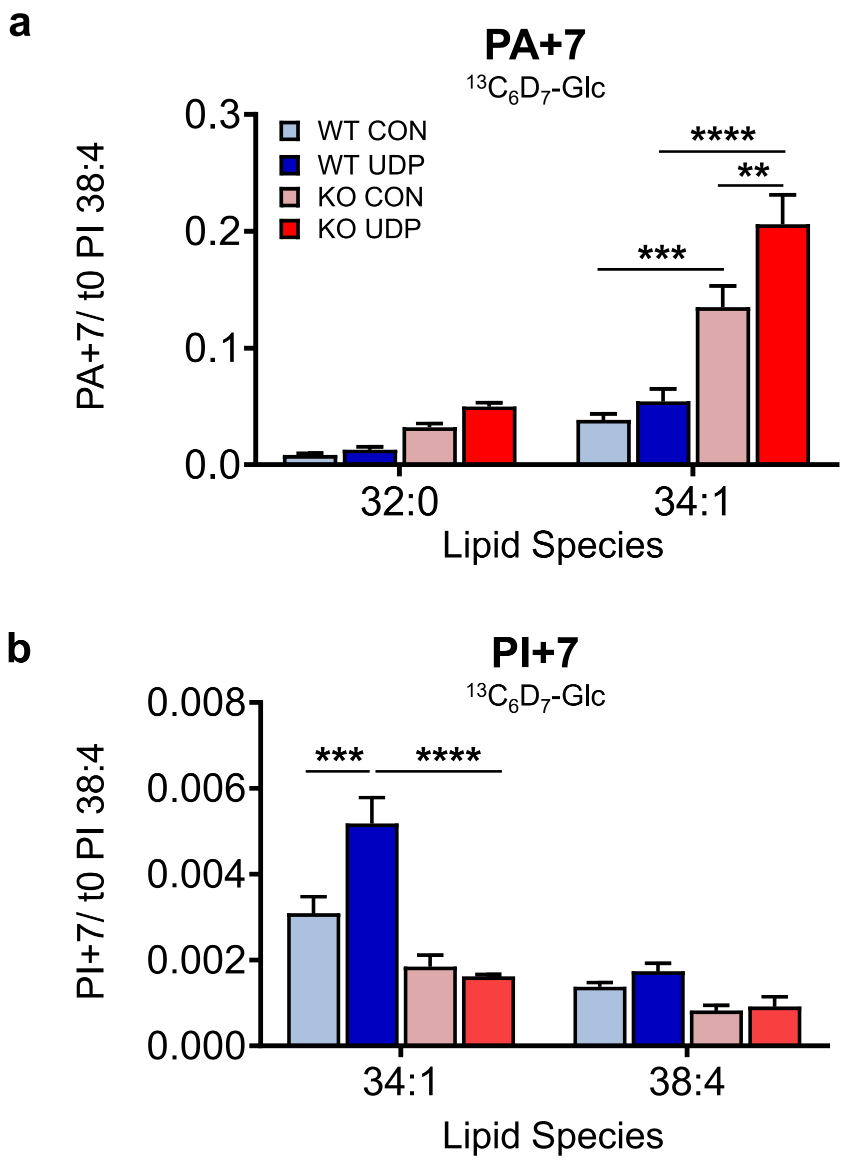

Figure S6. The effect of loss of CDS2 on a UDP-stimulated PI cycle

WT or CDS2-KO macrophages were labelled with $^{13}C_6D_7$-glucose for 30 min prior to incubation with or without 100μM UDP for 30min and then incorporation of +7 Da into the indicated isotopologues of PA and PI analysed by LC-MS/MS. Data are response ratios normalised to PI 38:4 at t=0 and represent means +/- S.E.M (n=3). Statistical significance was assessed using a *Two-Way ANOVA with Tukey's test* to correct for multiple comparisons (**p<0.01, ***p<0.001, ****p<0.0005).

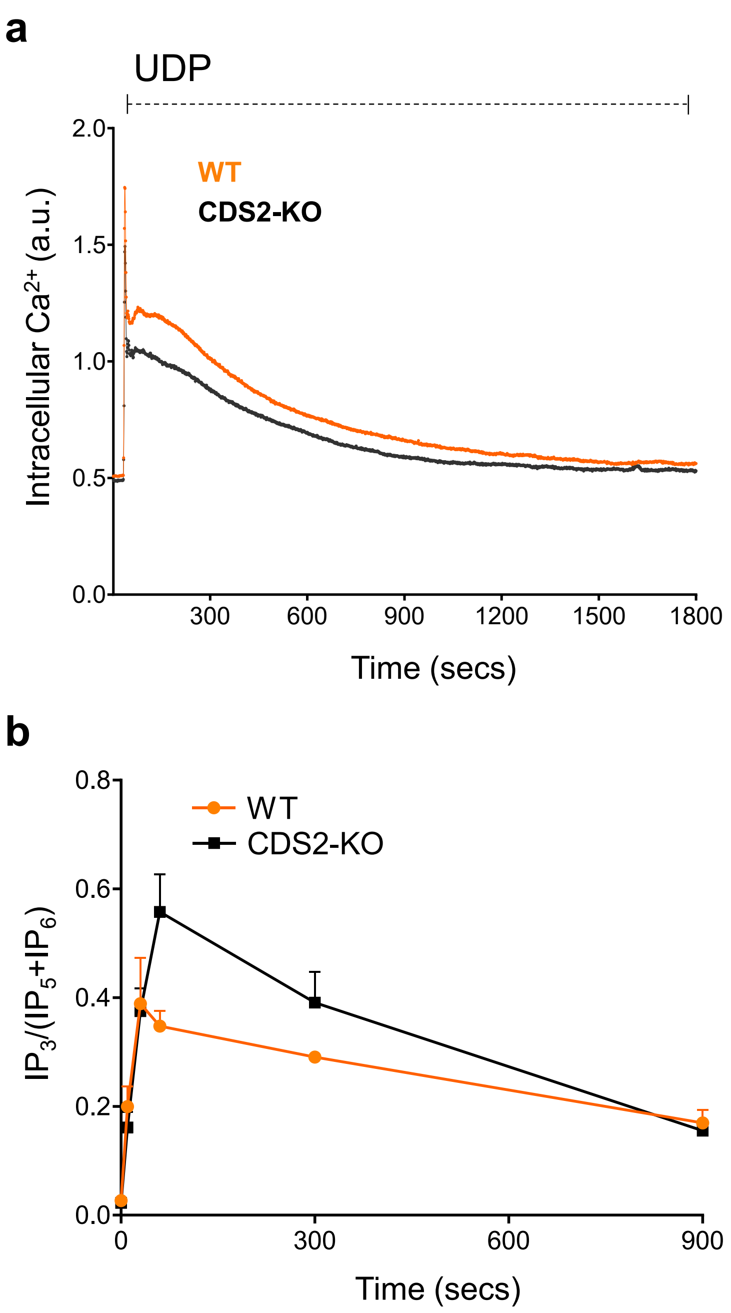

Figure S7. The effect of loss of CDS2 on a UDP-stimulated Ca^{2+} and $InsP_3$ responses.

a) WT or CDS2-KO macrophages were treated as described in Figure 10 and their intracellular Ca^{2+} concentration analysed by fluorescence imaging. Data are line traces representing the mean intracellular Ca^{2+} concentration from four independent biological experiments (as shown in Figure 10) but without the subtraction of the baseline value (average value before addition of UDP).

b) WT or CDS2-KO macrophages were treated with 100 μM UDP in Ca^{2+} -containing buffer for the indicated times, and inositol phosphates were extracted by perchloric acid and TiO_2 followed by analysis by CE-ESI-MS. Data are ion currents for $InsP_3$ normalised to the sum of the ion currents for $InsP_5$ and $InsP_6$ measured in the same samples, and are mean+/-S.E.M. from three independent experiments. Statistical significance was assessed for each paired timepoint using multiple paired t-test.

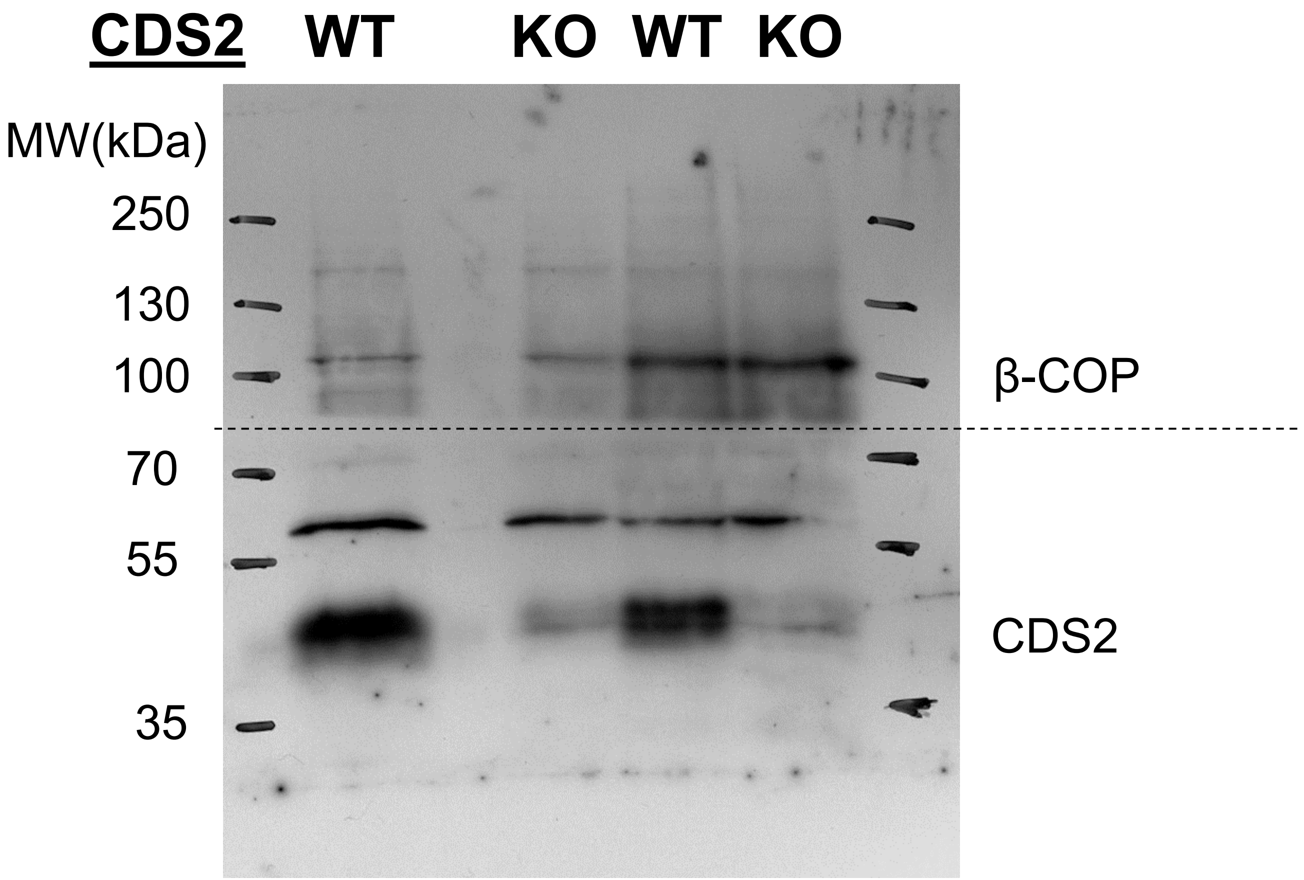

Figure S8. Supporting data for Figure S1.
Uncropped western blot for CDS2 and β-COP in WT and CDS2-KO mouse macrophages from which the cropped images in Figure S1 were derived. Following transfer of separated proteins, membranes were split by cutting between the 70 and 100kDa markers and the relevant parts of the membrane blotted independently for β-COP (top blot) or CDS2 (bottom blot). Bands on each membrane were detected by enchanced chemiluminescence and visualised on the same piece of film. Cropped images in FigureS1 result from the two right-hand lane samples. The additional left-hand lanes (not shown in the cropped image) are samples prepared by lysis in triton lysis buffer, rather than direct lysis in sample buffer as used for the final two lanes in FigureS1, and for other experiments as described in Materials and Methods.
